# Pathformer: a biological pathway informed transformer for disease diagnosis and prognosis using multi-omics data

**DOI:** 10.1093/bioinformatics/btae316

**Published:** 2024-05-13

**Authors:** Xiaofan Liu, Yuhuan Tao, Zilin Cai, Pengfei Bao, Hongli Ma, Kexing Li, Mengtao Li, Yunping Zhu, Zhi John Lu

**Affiliations:** MOE Key Laboratory of Bioinformatics, Center for Synthetic and Systems Biology, School of Life Sciences, Tsinghua University, Beijing 100084, China; Institute for Precision Medicine, Tsinghua University, Beijing 100084, China; MOE Key Laboratory of Bioinformatics, Center for Synthetic and Systems Biology, School of Life Sciences, Tsinghua University, Beijing 100084, China; Institute for Precision Medicine, Tsinghua University, Beijing 100084, China; MOE Key Laboratory of Bioinformatics, Center for Synthetic and Systems Biology, School of Life Sciences, Tsinghua University, Beijing 100084, China; MOE Key Laboratory of Bioinformatics, Center for Synthetic and Systems Biology, School of Life Sciences, Tsinghua University, Beijing 100084, China; Institute for Precision Medicine, Tsinghua University, Beijing 100084, China; MOE Key Laboratory of Bioinformatics, Center for Synthetic and Systems Biology, School of Life Sciences, Tsinghua University, Beijing 100084, China; Institute for Precision Medicine, Tsinghua University, Beijing 100084, China; MOE Key Laboratory of Bioinformatics, Center for Synthetic and Systems Biology, School of Life Sciences, Tsinghua University, Beijing 100084, China; Department of Rheumatology and Clinical Immunology, Peking Union Medical College Hospital, Chinese Academy of Medical Sciences, Peking Union Medical College, National Clinical Research Center for Dermatologic and Immunologic Diseases (NCRC-DID), MST State Key Laboratory of Complex Severe and Rare Diseases, MOE Key Laboratory of Rheumatology and Clinical Immunology, Beijing 100730, China; State Key Laboratory of Medical Proteomics, Beijing Proteome Research Center, National Center for Protein Sciences (Beijing), Beijing Institute of Lifeomics, Beijing 102206, China; MOE Key Laboratory of Bioinformatics, Center for Synthetic and Systems Biology, School of Life Sciences, Tsinghua University, Beijing 100084, China; Institute for Precision Medicine, Tsinghua University, Beijing 100084, China

## Abstract

**Motivation:**

Multi-omics data provide a comprehensive view of gene regulation at multiple levels, which is helpful in achieving accurate diagnosis of complex diseases like cancer. However, conventional integration methods rarely utilize prior biological knowledge and lack interpretability.

**Results:**

To integrate various multi-omics data of tissue and liquid biopsies for disease diagnosis and prognosis, we developed a biological pathway informed Transformer, Pathformer. It embeds multi-omics input with a compacted multi-modal vector and a pathway-based sparse neural network. Pathformer also leverages criss-cross attention mechanism to capture the crosstalk between different pathways and modalities. We first benchmarked Pathformer with 18 comparable methods on multiple cancer datasets, where Pathformer outperformed all the other methods, with an average improvement of 6.3%–14.7% in F1 score for cancer survival prediction, 5.1%–12% for cancer stage prediction, and 8.1%–13.6% for cancer drug response prediction. Subsequently, for cancer prognosis prediction based on tissue multi-omics data, we used a case study to demonstrate the biological interpretability of Pathformer by identifying key pathways and their biological crosstalk. Then, for cancer early diagnosis based on liquid biopsy data, we used plasma and platelet datasets to demonstrate Pathformer’s potential of clinical applications in cancer screening. Moreover, we revealed deregulation of interesting pathways (e.g. scavenger receptor pathway) and their crosstalk in cancer patients’ blood, providing potential candidate targets for cancer microenvironment study.

**Availability and implementation:**

Pathformer is implemented and freely available at https://github.com/lulab/Pathformer.

## 1 Introduction

Comparing to a single type of data, multi-omics data provide a more comprehensive view of gene regulation (Hasin *et al.* 2017). Therefore, integrating multi-omics data from tissue and liquid biopsies would be helpful in addressing challenges in disease diagnosis (Ning *et al.* 2023), treatment (Chiu *et al.* 2019, Sharifi-Noghabi *et al.* 2019), and prognosis (Hao *et al.* 2018), such as deregulated network between different types of molecules and data noise caused by patients’ heterogeneity (Tarazona *et al.* 2021). To integrate multi-omics data of cancer, several supervised methods have been developed, such as mixOmics (Rohart *et al.* 2017), liNN (Kuru *et al.* 2022), eiNN (Preuer *et al.* 2018), liCNN (Islam *et al.* 2020), eiCNN (Fu *et al.* 2020), MOGONet (Wang *et al.* 2021), and MOGAT (Xing *et al.* 2021). Later, the performance and interpretability of multi-omics data integration were further improved using deep learning models informed by biological pathways. For instance, a pathway-associated sparse deep neural network (PASNet) was utilized to predict the prognosis of glioblastoma multiforme (GBM) patients (Hao *et al.* 2018). Recently, P-NET, a sparse neural network integrating multiple molecular features based on a multilevel view of biological pathways, was introduced to predict subtype and survival of prostate cancer patients (Elmarakeby *et al.* 2021). In addition, PathCNN based on a convolutional neural network (CNN) was developed to predict the prognosis of GBM patients using principal component analysis (PCA) to define image-like multi-omics pathways (Oh *et al.* 2021).

These pathway-informed deep learning methods did not consider the crosstalk between omics and between pathways, although the crosstalk holds biological significance as well as pathway itself (Kim *et al.* 2007, Li *et al.* 2008, Prahallad and Bernards 2016, Liu *et al.* 2021). The crosstalk means a member of one pathway regulates a component of another pathway. The balance and oscillation between different pathways contribute to cancer progression and metastasis. For instance, a positive feedback loop between *Wnt* pathway and *ERK* pathway were revealed in cancer(Kim *et al.* 2007); crosstalk between *TGF-*β pathway and *TNF-*α pathway can promote tumor’s invasion and metastasis by affecting its microenvironment (Liu *et al.* 2021). Meanwhile, the criss-cross attention mechanism of the Transformer would be very useful to capture the crosstalk information (Jumper *et al.* 2021). However, incorporating multi-omics data and their crosstalk information in a Transformer is very challenging: when processing multi-omics data, the multi-modal features are usually multiplied by tens of thousands of genes, producing an extremely long input that is not acceptable by a common Transformer model (usually <512 words). Meanwhile, certain embedding methods for biological data, such as discretization and linear transformation, were introduced in the previous Transformer models (Osseni *et al.* 2022, Cui *et al.* 2023, Theodoris *et al.* 2023), while biological information was largely lost during these kinds of embedding.

In order to integrate multi-omics data by embedding biological pathway crosstalk without information loss, we introduce a Transformer model, Pathformer, with three key steps to address the above problems. First, it transforms various modalities into distinct gene-level features using a series of statistical methods, such as the maximum value method, and connects these features into a novel compacted multi-modal vector for each gene, which not only preserves valuable information but also shortens the input. Second, Pathformer utilizes a sparse neural network based on prior pathway knowledge to transform gene embeddings into pathway embeddings. Third, Pathformer naturally incorporates pathway crosstalk network into a Transformer model with bias to enhance the exchange of information between different pathways and between different modalities (e.g. omics) as well.

Here, we first benchmarked Pathformer and 18 other integration methods in various classification tasks, using multiple cancer tissue datasets from TCGA. Then, we used Pathformer to integrate various multi-omics data from tissue and liquid biopsies. Through case studies on survival prediction of breast cancer and noninvasive diagnosis of pan-cancer, we revealed interesting pathways, genes, and regulatory mechanisms related to cancer in human tissue and plasma, demonstrating the prediction accuracy and biological interpretability of Pathformer in various clinical applications.

## 2 Materials and methods

### 2.1 Overview of Pathformer

**Figure 1.**
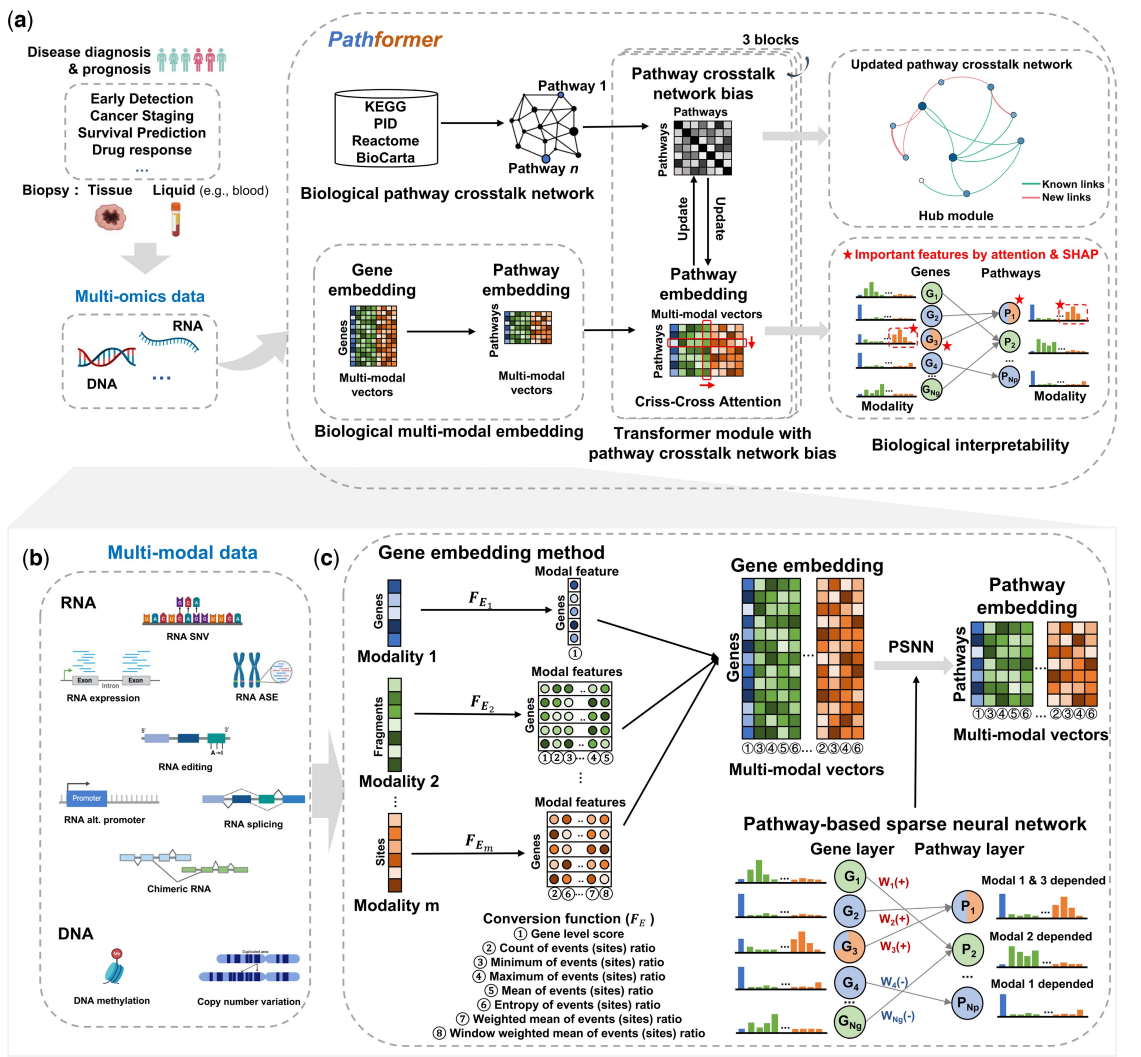
Overview of Pathformer. Schematic of Pathformer (a), which integrates multi-oimcs data of tissue and liquid biopsies for disease diagnosis and prognosis. Pathformer has six modules: (i) biological pathway and crosstalk network calculation module, (ii) multi-omics data input module (b), (iii) biological multi-modal embedding module (c), (iv) transformer module with pathway crosstalk network bias, (v) classification module, and (vi) biological interpretability module. $F_{E}$, conversion function in the gene embedding; G, gene; P, pathway; W, weight of pathway-based sparse neural network.

Pathformer is mainly designed to integrate various multi-omics data from tissue and liquid biopsies, which can be used for different classification tasks in disease diagnosis and prognosis, such as cancer early detection, cancer staging and survival prediction (Fig. 1a). It has six modules: (i) biological pathway and crosstalk network calculation module, (ii) multi-omics data input module, (iii) biological multi-modal embedding module (key module), (iv) Transformer module with pathway crosstalk network bias, (v) classification module, and (vi) biological interpretability module.

Pathformer combines prior biological pathway information (module 1, Fig. 1a) with multi-modal data (module 2, Fig. 1b) for disease diagnosis and prognosis. It introduces a new embedding method to incorporate biological multi-modal data at both gene level and pathway level: it initiates the process by uniformly transforming different modalities to the gene level through a series of statistical indicators, then concatenates these modalities into compacted multi-modal vectors to define gene embedding, and employs a sparse neural network based on the gene-to-pathway mapping to transform gene embedding into pathway embedding (module 3, Fig. 1c). Pathformer then enhances the fusion of information between various modalities and pathways by combining pathway crosstalk networks with Transformer encoder (module 4, Fig. 1a, Supplementary Fig. S1). Finally, a fully connected layer serves as the classifier for different downstream classification tasks (module 5). In addition, Pathformer uses a biological interpretable module with attention weights and SHapley Additive exPlanations (Lundberg and Lee 2017) values to identify important genes, pathways, modalities, and their crosstalk or regulation (module 6). These six modules are described in detail below.

### 2.2 Module 1: curation of biological pathways and calculation of initial crosstalk network

We curated 2289 biological pathways from four public databases including Kyoto Encyclopedia of Genes and Genomes database (KEGG) (Kanehisa and Goto 2000), Pathway Interaction database (PID) (Schaefer *et al.* 2009), Reactome database (Reactome) (Croft *et al.* 2010), and BioCarta Pathways database (BioCarta) (Nishimura 2001). Then, we filtered these pathways by three criteria: the gene number, the overlap ratio with other pathways (the proportion of genes in the pathway that are also present in other pathways), and the number of pathway subsets (the number of sub-pathways included in the pathway). Following the principle of moderate size and minimal overlap with other pathway information, we selected 1497 pathways with gene number between 15 and 100, or gene number >15 and overlap ratio <1, or gene number >15 and the number of pathway subsets <5. Next, we used *BinoX* (Ogris *et al.* 2017) to calculate the crosstalk relationship of pathways and build a pathway crosstalk network with adjacency matrix $P\in R^{N_{p}{\times N}_{p}}$, $N_{p}$ = 1497 (more details in Supplementary Note S1).

### 2.3 Modules 2 and 3: multi-omics data input and multi-modal embedding

Biological multi-modal data preprocessing and embedding method are two key modules of Pathformer (Fig. 1b and c). In module 2 (Fig. 1b), to capture more comprehensive regulatory information, we expanded biological multi-omics data into multi-modal data, including not only data from different omics sources but also variant features of the same omics, such as RNA splicing, RNA editing, RNA alternative promoter, and so on. To obtain multi-modal data, we used standardized bioinformatics pipeline to calculate different omics or variant features of the same omics from raw sequence reads (more details in Supplementary Note S2). These multi-modal data have different dimensions, including nucleotide level, fragment level, and gene level. For example, Pathformer’s input for cancer tissue datasets from Cancer Genome Atlas (TCGA) (Cancer Genome Atlas Research Network 2013) includes gene-level RNA expression, fragment-level DNA methylation, and both fragment-level and gene-level DNA CNV. Modalities and their dimension levels for different datasets are described in Supplementary Table S1.

In module 3 (Fig. 1c), we proposed a new biological multi-modal embedding method of Pathformer, which consists of gene embedding $E_{G}$ and pathway embedding $E_{P}$. We represented biological multi-modal input matrix of a sample as M, described as follows:

(1) $$M=\left[M_{1},M_{2},M_{3},\ldots ,M_{m}\right],\;M_{i}\in R^{N_{M_{i}}},\;i=1,\;2,\;3,\ldots ,m$$

where *m* is the number of modalities, and $N_{M_{i}}$ is the length of input for modality *i*, like the number of genes for RNA expression, the number of editing sites for RNA editing, and the number of CpG islands for DNA methylation.

Next, we first used a series of statistical indicators to convert different modalities into gene level modal features, and then concatenated these modal features into a compressed multi-modal vector as gene embedding $E_{G}\in R^{N_{g}\times D_{g}}$, which are calculated as follows:

(2) $$E_{G}=F_{E}\left(M\right)=\left[V_{1},\ldots ,V_{i},\ldots ,V_{m}\right]=\left[G_{1},\ldots ,G_{l},\ldots ,G_{N_{g}}\right]$$

(3) $$V_{i}=F_{E_{i}}\left(M_{i}\right)=\left[f_{*}\left(M_{i}\right),\ldots ,f_{*}\left(M_{i}\right)\right]=[V_{i}^{1},\ldots ,V_{i}^{e_{i}}]\in R^{N_{g}\times e_{i}}$$

(4) $$f_{*}\in [f_{1},\ldots ,f_{8}]$$

where $G_{l}=[V_{1}^{\left(l\right)},\ldots ,V_{i}^{\left(l\right)},\ldots ,V_{m}^{\left(l\right)}]{\in R}^{D_{g}}$ is gene embedding of the *l*th gene and is a compacted multi-modal vector; $V_{i}$ is the modal feature matrix of modality *i*; $V_{i}^{j}$ is the *j*th dimension of modal feature matrix for modality *i*; $N_{g}$ is the number of genes, $D_{g}=e_{1}+e_{2}+\cdots +e_{m}$ is the dimension of gene embedding; $e_{i}$ is the dimension of modal feature matrix for modality *i*; $F_{E}$ is the conversion function, which uses statistical indicators to uniformly convert different modalities into gene level; $F_{E_{i}}$ is the conversion function of modality *i*, and each modality’s function is constructed from distinct statistical indicator functions $f_{*}$ (more details in Supplementary Table S1). These statistical indicator functions include gene level score ($f_{1}$), count ($f_{2}$), minimum ($f_{3}$), maximum ($f_{4}$), mean ($f_{5}$), entropy ($f_{6}$), weighted mean in whole gene ($f_{7}$), and weighted mean in window ($f_{8}$), formulas of which are in Supplementary Note S3.

Subsequently, we used the known gene-pathway mapping relationship to develop a sparse neural network based on prior pathway knowledge (PSNN) to transform gene embedding $E_{G}$ into pathway embedding $E_{P}$, as described below:

(5) $$E_{P}={W_{\mathrm{sparse}}^{T}E}_{G}+B,\;E_{P}\in R^{N_{p}\times D_{p}}$$

where $N_{p}$ is the number of pathways, $D_{p}=D_{g}$ is the dimension of pathway embedding, $W_{\mathrm{sparse}}\in R^{N_{g}\times N_{p}}$ is a learnable sparse weight matrix, and B is a bias term. $W_{\mathrm{sparse}}$ is constructed based on the known relationship between pathways and genes. When the given gene and the pathway are irrelevant, the corresponding element of $W_{\mathrm{sparse}}$ will always be 0. Otherwise, it needs to be learned through training. Therefore, pathway embedding is a dynamic embedding method. The PSNN cannot only restore the mapping relationship between genes and pathways, but also capture the different roles of different genes in pathways, and can preserve the complementarity of different modalities. Additionally, this biological multi-modal embedding step does not require additional gene selection, thereby avoiding bias and overfitting problems resulting from artificial feature selection.

### 2.4 Module 4: transformer module with pathway crosstalk network bias

We developed the Transformer module based on criss-cross attention (CC-attention) with bias for data fusion of pathways, modalities, and their crosstalk (Supplementary Fig. S1). This module has 3 blocks, each containing multi-head column-wise self-attention (col-attention), multi-head row-wise self-attention (row-attention), layer normalization, GELU activation, residual connection, and network update. Particularly, col-attention is used to enhance the exchange of information between pathways, with the pathway crosstalk network matrix serving as the bias for col-attention to guide the flow of information. Row-attention is employed to facilitate information exchange between different modalities, and the updated pathway embedding matrix is used to update the pathway crosstalk network matrix by calculating the correlation between pathways.

Multi-head column-wise self-attention contains 8 heads, and each head is a mapping of $Q_{1}$,$K_{1}$,$V_{1}$,P, which are query vector, key vector, and value vector of pathway embedding $E_{P}$ and pathway crosstalk network matrix P, respectively. First, we represented the *h*th column-wise self-attention by $A_{\mathrm{col}}^{\left(h\right)}$, calculated as follows:

(6) $$Q_{1}=E_{P}W_{Q_{1}}^{\left(h\right)},\;K_{1}=E_{P}W_{K_{1}}^{\left(h\right)},\;V_{1}=E_{P}W_{V_{1}}^{\left(h\right)}$$

(7) $$A_{1}^{\left(h\right)}=(Q_{1}K_{1}^{T})/\sqrt{d}$$

(8) $$A_{\mathrm{col}}^{\left(h\right)}={\mathrm{dropout}}_{0.2}(\mathrm{softmax}(A_{1}^{\left(h\right)}+P))\cdot V_{1}^{\left(h\right)}$$

where h=1,2,…,H is the *h*th head; *H* is the number of heads; $W_{Q_{1}}^{\left(h\right)}\in R^{D_{p}\times d}$, $W_{K_{1}}^{\left(h\right)}\in R^{D_{p}\times d}$, $W_{V_{1}}^{\left(h\right)}\in R^{D_{p}\times d}$ are the weight matrices as parameters; *d* is the attention dimension; ${\mathrm{dropout}}_{0.2}$ is a dropout neural network layer with a probability of 0.2; and softmax is the normalized exponential function.

Next, we merged multi-head column-wise self-attention and performed a series of operations as follows:

(9) $$g_{1}^{\left(h\right)}=\mathrm{sigmoid}(E_{P}W_{g_{1}}^{\left(h\right)})$$

(10) $$U_{1}^{'}=U_{1}+E_{P},\;U_{1}=\sum_{h=1}^{H}(g_{1}^{\left(h\right)}\circ A_{\mathrm{col}}^{\left(h\right)}){\cdot W}_{U_{1}}^{\left(h\right)}$$

(11) $$O_{1}={\mathrm{dropout}}_{0.2}(\mathrm{GELU}(\mathrm{LN}(U_{1}^{'}){\cdot W}_{O_{11}}))\cdot W_{O_{12}}+U_{1}^{'}$$

where h=1,2,…,H is the *h*th head; *H* is the number of heads; ∘ is the matrix dot product operator; $W_{g_{1}}^{\left(h\right)}\in R^{D_{p}\times d}$, $W_{U_{1}}^{\left(h\right)}\in R^{d\times D_{p}}$, $W_{O_{11}}\in R^{D_{p}\times o}$, $W_{O_{12}}\in R^{o\times D_{p}}$ are the weight matrices as parameters; *o* is a constant; LN is the layer normalization function; GELU is the distortion of RELU activation function; and ${\mathrm{dropout}}_{0.2}$is a dropout neural network layer with a probability of 0.2.

Multi-head row-wise self-attention enables information exchange between different modalities. It is a regular dot-product attention. It also contains eight heads, and the *h*th row-wise self-attention, i.e. $A_{\mathrm{row}}^{\left(h\right)}$, is calculated as follows:

(12) $$Q_{2}=E_{P}^{T}W_{Q_{2}}^{\left(h\right)},\;K_{2}=E_{P}^{T}W_{K_{2}}^{\left(h\right)},\;V_{2}=E_{P}^{T}W_{V_{2}}^{\left(h\right)}$$

(13) $$A_{2}^{\left(h\right)}=(Q_{2}K_{2}^{T})/\sqrt{d}$$

(14) $$A_{\mathrm{row}}^{\left(h\right)}={\mathrm{dropout}}_{0.2}(\mathrm{softmax}(A_{2}^{\left(h\right)}))\cdot V_{2}^{\left(h\right)}$$

where h=1,2,…, h is the *h*th head; *H* is the number of heads; $W_{Q_{2}}^{\left(h\right)}\in R^{N_{p}\times d}$, $W_{K_{2}}^{\left(h\right)}\in R^{N_{p}\times d}$, $W_{V_{2}}^{\left(h\right)}\in R^{N_{p}\times d}$ are the weight matrices as parameters; *d* is the attention dimension; ${\mathrm{dropout}}_{0.2}$ is a dropout neural network layer with a probability of 0.2; and softmax is the normalized exponential function.

Subsequently, we merged multi-head row-wise self-attention and performed a series of operations. The formulas are as follows:

(15) $$g_{2}^{\left(h\right)}=\mathrm{sigmoid}(E_{P}^{T}W_{g_{2}}^{\left(h\right)})$$

(16) $$U_{2}^{'}={\beta *U}_{2}+E_{P}^{T},\;U_{2}=\sum_{h=1}^{H}(g_{2}^{\left(h\right)}\circ A_{\mathrm{row}}^{\left(h\right)}){\cdot W}_{U_{2}}^{\left(h\right)}$$

(17) $$O_{2}={\mathrm{dropout}}_{0.2}(\mathrm{GELU}(\mathrm{LN}(U_{2}^{'}){\cdot W}_{O_{21}}))\cdot W_{O_{22}}+U_{2}^{'}$$

where h=1,2,…, h is the *h*th head; *H* is the number of heads; ∘ is the matrix dot product operator; $W_{g_{2}}^{\left(h\right)}\in R^{N_{p}\times d}$, $W_{U_{2}}^{\left(h\right)}\in R^{d\times N_{p}}$, $W_{O_{21}}\in R^{N_{p}\times o}$, $W_{O_{22}}\in R^{o\times N_{p}}$ are the weight matrices as parameters; *o* is a constant; β is a constant coefficient for row-attention; LayerNorm is the layer normalization function; GELU is the distortion of RELU activation function; and ${\mathrm{dropout}}_{0.2}$ is a dropout neural network layer with a probability of 0.2. $O_{2}$ is pathway embedding input of the next Transformer block. In other words, when $E_{P}$ is $E_{P}^{\left(0\right)}$, $O_{2}$ is $E_{P}^{\left(1\right)}$. Superscripts with parenthesis represent data at different block.

Then, we used the updated pathway embedding $O_{2}$ to update the pathway crosstalk network. We exploited the correlation between embedding vectors of two pathways to update the corresponding element of the pathway crosstalk network matrix. The formula is as follows:

(18) $$P^{'}=(P\cdot P^{T})/N_{p}$$

where $P^{\prime }$ is the updated pathway crosstalk network matrix of the next Transformer block. In other words, when $P^{\prime }$ is $P^{\left(1\right)}$, P is $P^{0}$. Superscripts with parenthesis represent data at different block.

### 2.5 Module 5: classification module

Given the classification tasks in disease diagnosis and prognosis, we used the fully connected neural network as the classification module to transform pathway embedding encoded by the Transformer module into the probability for each label. Three fully connected neural networks each have 300, 200, and 100 neurons, with dropout probability ${\mathrm{dropout}}_{c}$, which is a hyperparameter. More details are described in Supplementary Note S4.

### 2.6 Module 6: biological interpretability module

The biological interpretable module enables us to calculate the contribution of each modality, identify important pathways and their key genes, and uncover the most critical pathway crosstalk subnetworks.

To calculate the contribution of each omics and each modality, we first integrated all matrices of row-attention maps into one matrix by element-wise averaging. Then, we averaged this average row-attention matrix along with columns as the attention weights of modalities. More details are described in Supplementary Note S5.

To identify important pathways and their key genes, we used SHapley Additive exPlanations (Lundberg and Lee 2017) (SHAP value) to calculate the contribution of each feature. It is an additive explanation model inspired by coalitional game theory, which regards all features as “contributors.” SHAP value is the value assigned to each feature, which explains the relationship between modalities, pathways, genes and classification, implemented by “SHAP” package of *Python* v3.6.9. Then, pathways with the top 15 SHAP values in the classification task are considered as important pathways. For each pathway, genes with top five SHAP values are considered as the key genes of the pathway. The modality of a gene with the rank of SHAP value higher than other modalities is considered the core modality of the gene. More details are described in Supplementary Note S5.

Particularly, the pathway crosstalk network matrix is used to guide the direction of information flow, and updated according to updated pathway embedding in each Transformer block. Therefore, the updated pathway crosstalk network contains not only the prior information in the initial network (module 1) but also the multi-modal data information derived from the Transformer module (module 4), which represents the specific regulatory mechanism in each classification task. We defined the sub-network score through SHAP value of each pathway in the sub-network, so as to find foremost sub-network for prediction, i.e. hub module of the updated pathway crosstalk network. The calculation of the sub-network score can be divided into four steps: average pathway crosstalk network matrix calculation, network pruning, sub-network boundary determination, and score calculation. More details of sub-network score calculations are described in Supplementary Note S5.

### 2.7 Experimental settings

#### 2.7.1 Data collection and preprocessing

We assayed both tissue biopsy and liquid biopsy data in this study. First, for benchmark testing on cancer diagnosis and prognosis, we collected multiple datasets of different cancer types from TCGA (tissue data) to evaluate the classification performance, including 10 datasets for early- and late-stage classification, 10 datasets for low- and high-risk survival classification, and 5 datasets for drug responses prediction (Supplementary Fig. S2). In addition, we also collected and processed two types of body fluid datasets: the plasma dataset [373 samples assayed by total cell-free RNA-seq (Chen *et al.* 2022, Tao *et al.* 2023)] and the platelet dataset [918 samples assayed by blood platelet RNA-seq (Best *et al.* 2015, Best *et al.* 2017)]. Through our biological information pipeline, 3 and 7 biological modalities were derived from the TCGA (tissue biopsy) datasets and the liquid biopsy datasets, respectively. More details are described in Supplementary Notes S2.

#### 2.7.2 Model training and test

We implemented Pathformer’s network architecture using the “PyTorch” package in *Python* v3.6.9, and our codes can be found in the GitHub repository (https://github.com/lulab/Pathformer). For model training and test, we used 5-fold cross-validation, and repeated it twice by shuffling. Before evaluating the performance on test sets, we optimized hyperparameters (e.g. learning rate, dropout probability of classification and constant coefficient for row-attention) and epoch numbers inside the training set only. More details of model training and test are described in Supplementary Note S6.

#### 2.7.3 Evaluation criteria

When evaluating the classification performance, we used at least three evaluation indicators, area under the receiver operating characteristic curve (AUC), weighted-averaged F1 score (F1score_weighted), and macro-averaged F1 score (F1score_macro). Notably, we prioritized F1score_macro as the main evaluation criterion in this paper. This choice stems from the imbalance of sub-classes in our data, where F1score_macro stands out as a fairer and more robust indicator compared to other metrics such as AUC.

## 3 Results

### 3.1 Benchmark of Pathformer and 18 multi-omics data integration methods using TCGA data

**Figure 2.**
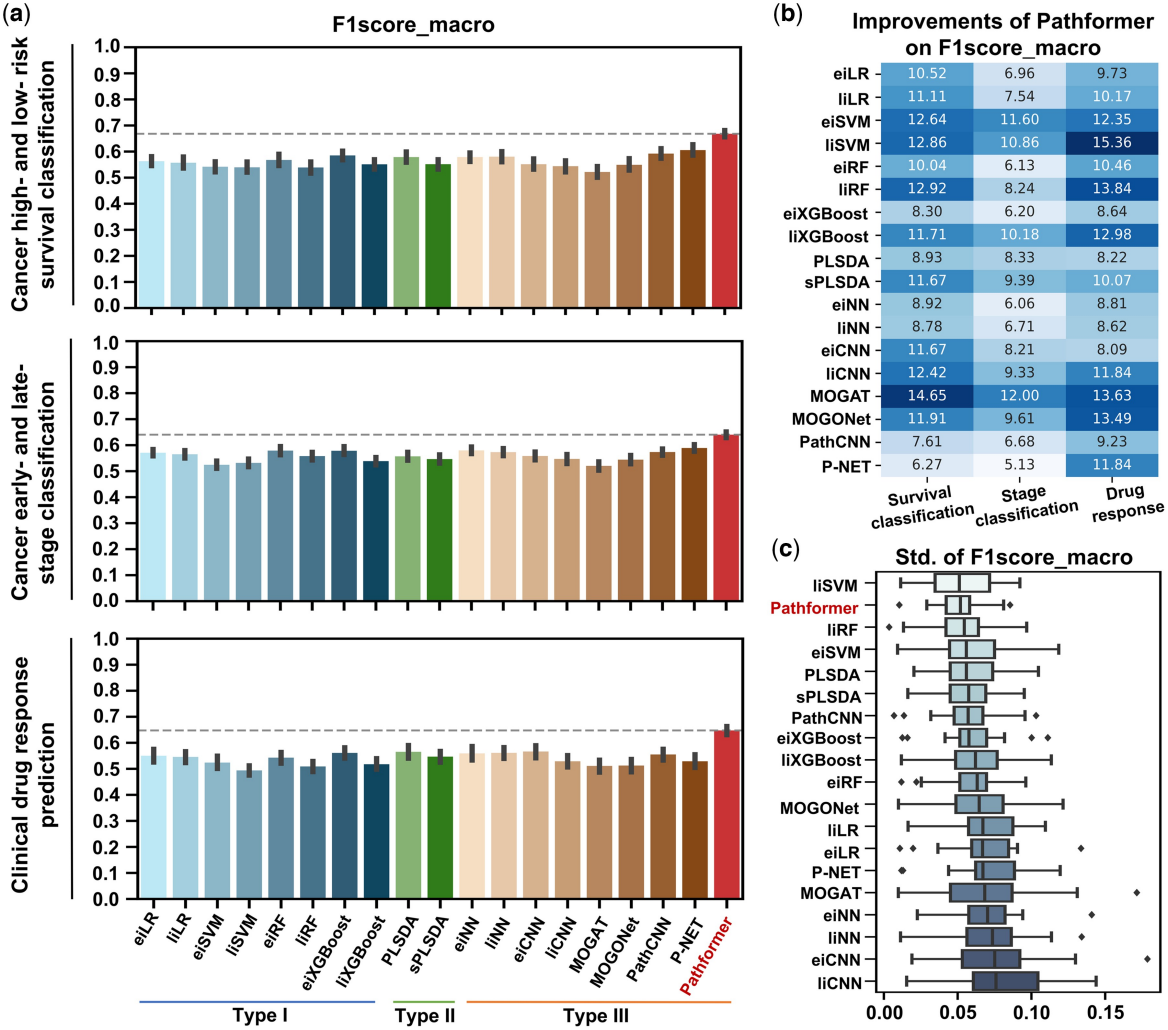
Performance comparison among multiple multi-omics data integration methods. Average macro-averaged F1 score (a), its percentage gap from Pathformer (b), and its standard deviation (c) are shown for each method on the TCGA datasets (all cancer types) for cancer low- and high-risk survival classification, early- and late-stage classification, and clinical drug response prediction, respectively. Error bars are from 5-fold cross-validation repeated twice (10 values) of all datasets.

We conducted a meticulous benchmark of Pathformer and 18 other multi-omics integration methods for various classification tasks in cancer diagnosis, treatment, and prognosis (Fig. 2). These methods can be categorized into three types. Type I includes early and late integration methods based on conventional classifiers, such as support vector machine (SVM), logistic regression (LR), random forest (RF), and extreme gradient boosting (XGBoost). Type II includes partial least squares-discriminant analysis (PLSDA) and sparse partial least squares-discriminant analysis (sPLSDA) of mixOmics (Rohart *et al.* 2017). Type III consists of deep learning-based integration methods, i.e. eiNN (Preuer *et al.* 2018), liNN (Kuru *et al.* 2022), eiCNN (Fu *et al.* 2020), liCNN (Islam *et al.* 2020), MOGONet (Wang *et al.* 2021), MOGAT (Xing *et al.* 2021), P-NET (Elmarakeby *et al.* 2021) and PathCNN (Oh *et al.* 2021). Among these, eiNN and eiCNN are early integration methods based on NN and CNN; liNN and liCNN are late integration methods based on fully connected neural network (NN) and convolutional neural network (CNN); MOGONet and MOGAT are multi-modal integration methods based on graph neural network; P-NET and PathCNN are representative multi-modal integration methods that combines pathway information. More details of comparison methods are in Supplementary Note S7.

To evaluate the performance, we tested the methods on multiple TCGA datasets for three tasks: cancer survival prediction, cancer staging, and drug response prediction. DNA methylation, DNA copy number variation (CNV), and RNA expression were used as input. Optimal hyperparameter combination for each dataset are listed in Supplementary Table S2. Considering the imbalanced numbers of sub-classes in the TCGA data, we utilized the macro-averaged F1 score as the primary evaluation metric for hyperparameter optimization and performance evaluation (Fig. 2 and Tables 1). Other evaluation indicators (e.g. AUC) are listed in Supplementary Table S3.

**Table 1.** Performance comparison among multiple multi-omics data integration methods for TCGA datasets.

	Methods	Type I								Type II	
		eiLR	eiRF	eiSVM	eiXGBoost	liLR	liRF	liSVM	liXGBoost	PLSDA	sPLSDA
**Survival classification** ^a^	BRCA^d^	0.571	0.494	0.467	0.564	0.569	0.473	0.468	0.523	0.561	0.549
	KIRC	0.584	0.636	0.656	0.653	0.619	0.622	0.614	0.585	0.660	0.617
	LUAD	0.530	0.443	0.487	0.530	0.479	0.453	0.438	0.481	0.444	0.450
	LUSC	0.520	0.477	0.471	0.519	0.481	0.452	0.508	0.491	0.496	0.495
	HNSC	0.529	0.497	0.466	0.476	0.512	0.478	0.470	0.483	0.527	0.521
	BLCA	0.479	0.475	0.459	**0.549**	0.456	0.438	0.475	0.504	0.506	0.465
	LIHC	0.497	0.579	0.436	0.550	0.487	0.428	0.439	0.500	0.514	0.442
	SKCM	0.557	0.609	0.630	0.594	0.554	0.609	0.561	0.532	0.635	0.593
	LGG	0.689	0.759	0.640	0.723	0.718	0.724	0.714	0.715	**0.778**	0.726
	Pan-cancer^e^	0.674	0.709	0.705	0.694	0.696	0.712	0.710	0.697	0.669	0.658
**Stage classification** ^b^	BRCA	0.510	0.488	0.451	0.518	0.522	0.454	0.436	0.475	0.456	0.450
	KIRC	0.647	**0.723**	0.661	0.686	0.654	0.704	0.675	0.670	0.710	0.717
	LUAD	0.506	0.503	0.460	0.526	0.473	0.497	0.460	0.471	0.462	0.503
	LUSC	0.501	0.509	0.467	0.498	0.506	0.469	0.470	0.483	0.487	0.465
	STAD	0.541	0.540	0.528	0.548	**0.581**	0.543	0.552	0.493	0.565	0.540
	BLCA	0.612	0.647	0.555	0.639	0.624	0.611	0.564	0.542	0.609	0.568
	LIHC	0.567	0.528	0.464	0.540	0.496	0.507	0.438	0.526	0.521	0.552
	SKCM	0.579	0.571	0.551	0.535	0.548	0.570	0.557	0.516	0.562	0.514
	THCA	0.613	0.660	0.550	**0.664**	0.626	0.622	0.601	0.601	0.627	0.596
	Pan-cancer^f^	0.631	0.622	0.557	0.629	0.620	0.604	0.567	0.608	0.572	0.559
**Drug response Prediction** ^c^	Carboplatin^g^	0.559	0.564	0.541	0.587	0.541	0.525	0.513	0.543	0.553	0.556
	Cisplatin	0.577	0.564	0.507	0.564	0.525	0.482	0.444	0.543	0.568	0.566
	Fluorouracil	0.488	0.506	0.466	0.524	**0.528**	0.459	0.484	0.460	0.509	0.444
	Gemcitabine	0.553	0.552	0.556	0.540	0.533	0.546	0.532	0.542	0.606	**0.617**
	Paclitaxel	0.574	0.529	0.549	0.591	**0.602**	0.533	0.496	0.501	0.591	0.551

	Methods	Type III								Pathformer
		eiNN	eiCNN	liNN	liCNN	MOGONet	MOGAT	PathCNN	P-NET	Pathformer
**Survival classification** ^a^	BRCA^d^	0.573	0.510	0.576	0.536	0.510	0.466	0.558	**0.640**	**0.673**
	KIRC	0.640	0.548	0.631	0.596	0.632	0.637	0.643	**0.661**	**0.688**
	LUAD	0.503	0.462	0.521	0.504	0.455	0.438	0.522	**0.551**	**0.633**
	LUSC	0.483	0.529	0.480	0.501	0.497	0.434	**0.560**	0.509	**0.615**
	HNSC	0.537	0.512	0.523	0.487	0.469	0.462	0.525	**0.559**	**0.606**
	BLCA	0.497	0.474	0.518	0.460	0.470	0.443	0.486	0.470	**0.601**
	LIHC	0.593	0.578	0.551	0.465	0.476	0.448	**0.598**	0.578	**0.651**
	SKCM	0.583	0.582	0.590	0.489	0.599	0.605	0.551	**0.641**	**0.694**
	LGG	0.695	0.631	0.702	0.706	0.706	0.595	0.766	0.715	**0.787**
	Pan-cancer^e^	0.687	0.688	0.713	0.697	0.676	0.689	0.712	**0.733**	**0.735**
**Stage classification** ^b^	BRCA	**0.545**	0.522	0.535	0.518	0.466	0.463	0.525	0.522	**0.573**
	KIRC	0.669	0.647	0.681	0.643	0.646	0.637	0.694	0.624	**0.726**
	LUAD	0.549	**0.563**	0.531	0.559	0.461	0.493	0.554	0.543	**0.629**
	LUSC	0.515	0.501	0.528	0.473	0.476	0.459	**0.534**	0.526	**0.564**
	STAD	0.555	0.505	0.518	0.482	0.574	0.562	0.521	0.537	**0.596**
	BLCA	0.636	0.634	0.616	0.565	0.553	0.554	0.566	**0.660**	**0.706**
	LIHC	0.569	0.584	0.565	0.586	0.474	0.461	0.563	**0.612**	**0.616**
	SKCM	**0.614**	0.553	0.582	0.557	0.526	0.528	0.54	0.571	**0.646**
	THCA	0.528	0.469	0.547	0.468	0.644	0.574	0.595	0.632	**0.690**
	Pan-cancer^f^	0.617	0.606	0.630	0.618	0.624	0.473	0.643	**0.664**	**0.657**
**Drug response Prediction** ^c^	Carboplatin^g^	0.589	0.588	0.556	0.565	0.504	0.504	**0.602**	0.581	**0.680**
	Cisplatin	0.555	0.593	**0.608**	0.574	0.469	0.525	0.575	0.531	**0.652**
	Fluorouracil	0.482	0.512	0.495	0.492	0.500	0.442	0.486	0.489	**0.602**
	Gemcitabine	0.588	0.565	0.564	0.528	0.585	0.558	0.549	0.506	**0.721**
	Paclitaxel	0.583	0.575	0.583	0.487	0.504	0.527	0.564	0.539	**0.660**

a Ten TCGA datasets of survival classification are tested. Average macro-averaged F1 scores are listed for the two unbalanced classes, high- and low-risk survival cancer patients. Each value is the mean of 5-fold cross-validation repeated twice (10 values).

b Ten TCGA datasets of stage classification are tested. Average macro-averaged F1 scores are listed for the two unbalanced classes, early- (stage I and II) and late-stage (stage III and IV) cancer patients. Each value is the mean of 5-fold cross-validation repeated twice (10 values).

c Five drug response datasets from TCGA are tested. Average macro-averaged F1 scores are listed for the two unbalanced classes, responder (including complete response and partial response) and nonresponder (including stable disease and progressive disease) from cancer patients. Each value is the mean of 5-fold cross-validation repeated twice (10 values).

d Abbreviation of cancer type according to the TCGA terms.

e Pan-cancer dataset of survival classification contains 33 cancer types of TCGA terms.

f Pan-cancer dataset of stage classification contains 21 cancer types of TCGA terms.

g Abbreviation of drug type.

The bold values are within the top two in each dataset.

In general, Pathformer significantly performed better than 18 other integration methods in terms of F1score_macro score (Fig. 2a and b) and cross-validation variances (Fig. 2c). In cancer low- and high-risk survival classification tasks, comparing to the other eight deep learning methods (Type III), Pathformer’s F1score_marco showed average improvements between 6.3% and 14.6%. When comparing to eiXGBoost, which performed best in the conventional machine learning methods (Types I and II), Pathformer’s F1score_marco showed an average improvement of 8.3% (Fig. 2b). In early- and late-stage classification tasks, comparing to the deep learning methods (Type III), Pathformer’s F1score_marco showed average improvements between 5.1% and 12%. Compared to eiXGBoost, Pathformer’s F1score_marco showed an average improvement of 6.2% (Fig. 2b). In drug response prediction tasks, comparing to the deep learning methods (Type III), Pathformer’s F1score_marco showed average improvements between 8% and 13.6%. When comparing to eiXGBoost, Pathformer’s F1score_marco showed an average improvement of 8.6% (Fig. 2b). Moreover, Pathformer demonstrated reduced variance (Fig. 2c) and a stronger correlation between predictive confidence scores and fraction of positives (Supplementary Fig. S7) in cross-validation, indicating greater stability and reliability.

The detailed performance comparisons of Pathformer and other integration methods for different cancer types are shown in Tables 1 and Supplementary Table S3. In survival classifications, Pathformer achieved the highest F1score_macro and F1score_weighted in all the 10 datasets, and the highest AUC in 7 of 10 datasets. In stage classifications, Pathformer achieved the highest F1score_macro in 9 of 10 datasets, the highest F1score_weighted in 8 of 10 datasets, and the highest AUC in 6 of 10 datasets. In drug response prediction, Pathformer achieved the highest F1score_macro, F1score_weighted and AUC in all datasets.

### 3.2 Ablation analysis of Pathformer

**Figure 3.**
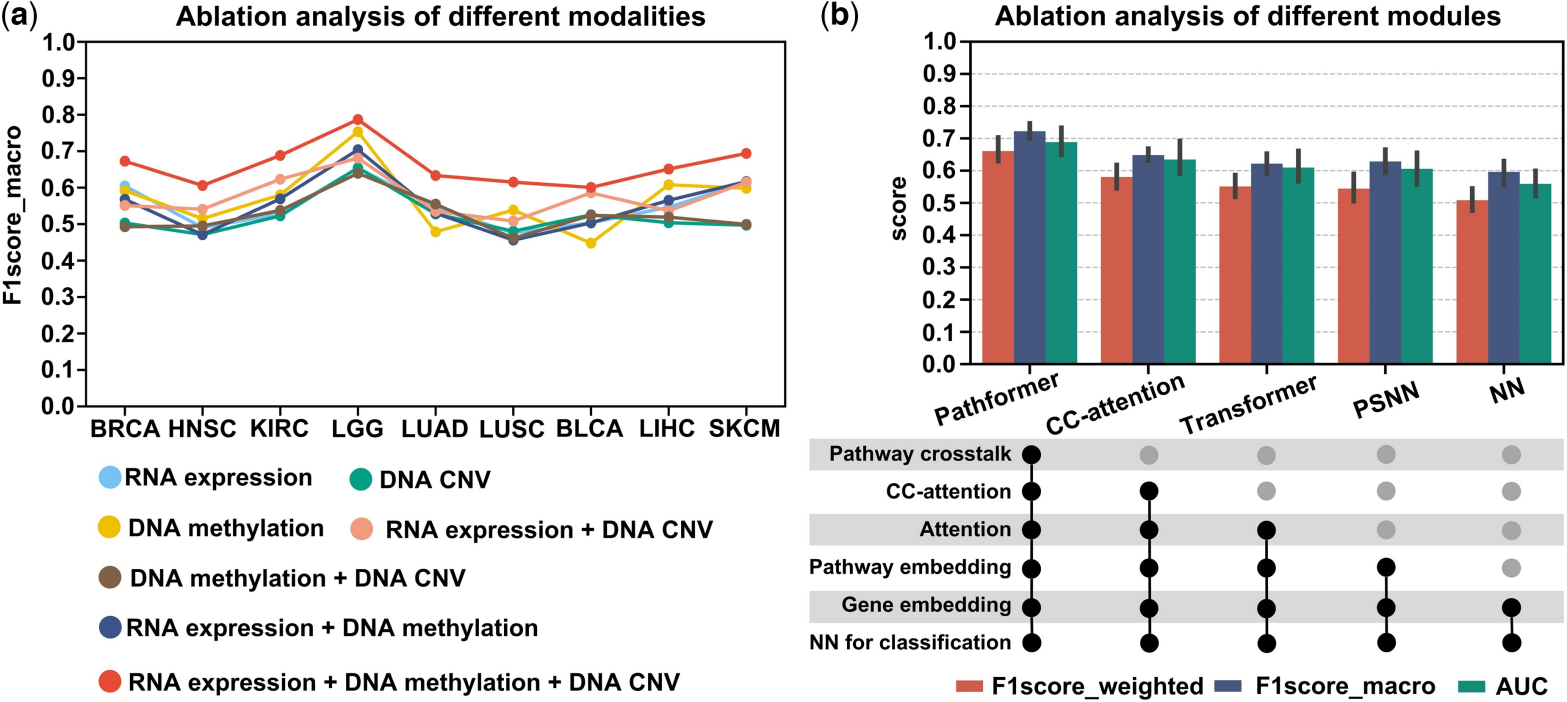
Ablation analysis of Pathformer for different input modalities and different calculation modules. (a) Different types of input modalities (omics data types) were used as input for TCGA cancer low- and high-risk survival classification. (b) Ablation analysis of different calculation modules in Pathformer. Error bars are from 2 times 5-fold cross-validation across 9 datasets, representing 95% confidence intervals. CC-attention, Pathformer without pathway crosstalk network bias; Transformer, Pathformer with only normal attention and pathway embedding; PSNN, Pathformer with only classification module with pathway embedding; NN, Pathformer with only classification module with gene embedding.

We used ablation analysis to evaluate the contributions of different input modalities and calculation modules in the Pathformer model, based on nine datasets for cancer survival prediction (Fig. 3), nine datasets for cancer stage classification (Supplementary Fig. S8), and five datasets for drug response prediction (Supplementary Fig. S9). The pan-cancer dataset in cancer survival and stage classification was not used here. Firstly, to evaluate the contribution of integrating different modalities of data to classification, we compared seven models, including Pathformer with three modalities as input (RNA expression + DNA methylation + DNA CNV), Pathformer with two modalities as input (RNA expression + DNA methylation, RNA expression + DNA CNV, and DNA methylation + DNA CNV), and Pathformer with a single modality as input (RNA expression-only, DNA methylation-only, and DNA CNV-only). By comparing the performances of these models on cancer survival risk classification, we discovered that the model with all three modalities as input achieved the best performance, followed by the model with RNA expression and DNA CNV, and the model with DNA methylation-only (Fig. 3a). Furthermore, we observed that the performances of models with single modality as input can vary greatly between datasets. For example, DNA methylation-only model performed better than RNA expression-only and DNA CNV-only model in the LUSC, LIHC, and LGG datasets, but the opposite results were observed in the LUAD and BLCA datasets. Ablation analysis of different modalities on cancer stage classification (Supplementary Fig. S8a) and drug response prediction (Supplementary Fig. S9a) showed similar results. These findings underscore the distinct behaviors of different modalities in different cancer types, highlighting the necessity of multi-modal data integration in various cancer stage and survival risk classification tasks.

Next, to evaluate the essentialities of different calculation modules in Pathformer, we compared four additional variations of Pathformer, namely CC-attention, Transformer, PSNN, and NN, in which one to multiple modules of Pathformer are successively removed. The “CC-attention” model is Pathformer without pathway crosstalk network bias. The “Transformer” model is Pathformer without pathway crosstalk network bias and row-attention, using only normal attention mechanism and pathway embeddings. The “PSNN” model directly uses classification module with pathway embedding as input. The “NN” model directly uses classification module with gene embedding as input. As shown in Fig. 3b and Supplementary Figs S8 and S9, the complete Pathformer achieved the best classification performance, while the performance of CC-Attention, Transformer, PSNN, and NN decreased successively. This indicates that pathway crosstalk network, attention mechanism, and pathway embedding are all integral components of Pathformer. In particular, CC-attention exhibited significantly poorer classification performance compared to Pathformer, providing strong evidence for the necessity of incorporating pathway crosstalk in Pathformer.

### 3.3 Biological interpretability of Pathformer in breast cancer prognosis prediction using tissue data

**Figure 4.**
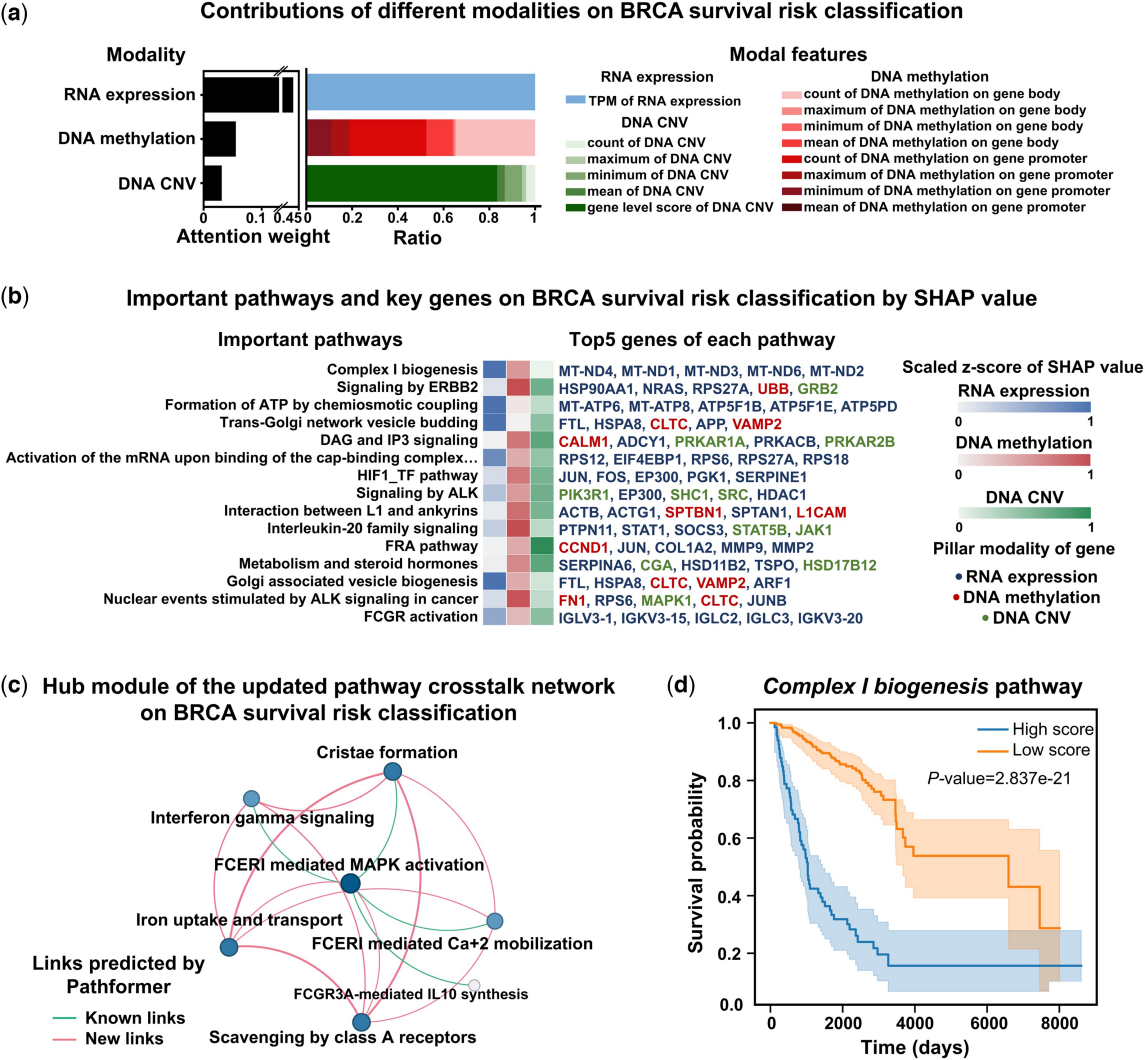
Biological interpretation of the breast cancer survival data using Pathformer. (a) Contributions of different modalities for breast cancer (BRCA) survival risk classification calculated by attention weights (averaging attention maps of row-attention). (b) Important pathways and their key genes with top SHapley Additive exPlanations (SHAP) values for BRCA survival risk classification. Among the key genes, different colors represent different pillar modalities of the genes. (c) A hub module of the updated pathway crosstalk network for BRCA survival risk classification. Color depth and size of node represents the degree of node. Line thickness represents the weight of edge. All links are predicted by Pathformer, where known links are reported by the initial crosstalk network and new links are new predictions. (d) Keplan–Meier curves of the most active pathway selected identified by Pathformer. *P*-value calculated through Log-Rank test.

To further understand the decision-making process of Pathformer and validate the reliability of its biological interpretability, we showed a case study on breast cancer survival risk classification. We demonstrated that Pathformer can use attention weights and SHAP values to identify modalities, pathways, and genes statistically associated with breast cancer prognosis, which aligns with known biological knowledge (Fig. 4).

First, at the omics and modality level, we visualized the contributions of different modalities for breast cancer survival risk classification by the attention weights (Fig. 4a). The contribution of transcriptomic data was the greatest in breast cancer prognostic prediction, which is consistent with the results of ablation analysis (Fig. 3a) and findings from other literatures (Huang *et al.* 2019, Tong *et al.* 2021). Additionally, from Fig. 4a and Supplementary Fig. S10a, we observed that the contribution of various features in the same modality varied between BRCA prognosis and staging, such as DNA methylation. These findings further validate the necessity of biological multi-modal embedding and integration.

Next, at the pathway and gene levels, we identified key pathways with top 15 SHAP values and key genes with top 5 SHAP values for each pathway in breast cancer survival risk classification (Fig. 4b). Then, we presented a hub module of the updated pathway crosstalk network (Fig. 4c). These key pathways and genes identified by SHAP and the hub module are biologically meaningful and consistent with previous biological experiments. For instance, *complex I biogenesis* pathway, which was identified as the most critical pathway during the classification and a key node in the hub module of the updated pathway crosstalk network, was reported to play an important role in cancer cell proliferation and metastasis (Urra *et al.* 2017). Five mitochondrial genes (MT-ND4, MT-ND1, MT-ND3, MT-ND6, and MT-ND2), which were identified as key genes of the *complex I biogenesis* pathway by Pathformer, were also reported to be associated with breast cancer prognosis (Kopinski *et al.* 2021). Another example is FTL, which was predicted by our SHAP value to be up-regulated in the high-risk cancer group, was also reported to promote breast cancer cell proliferation validated by knockout experiments (Tang *et al.* 2023).

Subsequently, to facilitate a more intuitive understanding of the impact of active pathways identified by Pathformer on breast cancer survival risk classification, we depicted survival curves comparing patients with high and low scores in active pathways (Fig. 4d and Supplementary Fig. S11). The pathway score for each sample was obtained by averaging across different dimensions of pathway embedding updated by Pathformer. Log-rank tests indicated that most active pathways identified by Pathformer, like *complex I biogenesis* pathway, significantly influenced patient survival. Finally, to gain further insights into how Pathformer uses key features for accurate decision-making, we visualized pathway embedding changes and discussed the commonalities among correctly classified samples (Supplementary Note S9). We visually examined the feature extraction capability of Pathformer’s Transformer module with CC-attention by comparing PCA of pathway embedding matrices before and after Pathformer update, and pathway score heatmap (Supplementary Fig. S12a and b). We also found that Pathformer’s accuracy is not influenced by clinical indicators such as cancer subtype and age (Supplementary Fig. S12c).

### 3.4 Performance of Pathformer for the noninvasive diagnosis of cancer using liquid biopsy data

In clinical practice, cancer diagnosis involves not only using tissue data for cancer staging but also using liquid biopsy data (i.e. plasma) for noninvasive early detection and screening. The latter has even greater clinical significance because early detection substantially increases five-year survival rate of cancer patients. For instance, 5-year survival rates of colon cancer were reported as 93.2% for stage I, and only 8.1% for stage IV (O’Connell *et al.* 2004). Therefore, we applied Pathformer to liquid biopsy data, aiming to classify cancer patients from healthy controls. We curated two types of cell-free RNA sequencing (cfRNA-seq) data, including plasma datasets (comprising 98 healthy donors and 275 cancer samples) and platelet datasets (comprising 286 healthy donors and 632 cancer samples). We then calculated seven RNA-level modalities as Pathformer’s multi-modal input, including RNA expression, RNA splicing, RNA editing, RNA alternative promoter (RNA alt. promoter), RNA allele-specific expression (RNA ASE), RNA single nucleotide variations (RNA SNV), and chimeric RNA. Liquid biopsy data collection and preprocessing procedures are in Supplementary Note S2, while model parameters and settings are in Supplementary Note S6. Because these seven modalities of RNA may have information redundancy, we selected the best modality combination based on 2 times 5-fold cross validations (Supplementary Note S10). The results showed that the plasma data with seven modalities and the platelet data with three modalities obtained the best performances (AUCs > 0.9). Additionally, we found that Pathformer’s performance was superior to the other integration methods using the liquid biopsy data (Tables 2 and Supplementary Table S4). Because cancer screening usually requires high specificity, we particularly report sensitivities on 99% specificity in Table 2. Pathformer achieves an average sensitivity of 48.8% in the plasma dataset and an average sensitivity of 48.1% in the platelet dataset. It is worth noting that the sensitivity is still above 45% on 99% specificity in the plasma data even for the early-stage cancer patients, showing Pathformer’s potential for early cancer diagnosis.

**Table 2.** Cancer detection performance of Pathformer and other integration methods based on the cell-free RNA liquid biopsy data.

Methods		Dataset	Macro-averaged F1 score	Weighted-averaged F1 score	AUC	Sensitivity (99% specificity)
Type I	eiLR	Plasma^a^	0.795	0.840	0.875	0.316
	eiXGBoost	Plasma	0.777	0.831	0.869	0.324
	eiSVM	Plasma	0.814	0.861	0.910	0.367
	eiRF	Plasma	0.792	0.847	0.882	0.370
	liSVM	Plasma	0.641	0.754	0.904	0.431
	liRF	Plasma	0.754	0.823	0.897	0.438
	liLR	Plasma	0.698	0.788	0.910	0.462
	liXGBoost	Plasma	0.790	0.845	0.911	0.467
Type II	PLSDA	Plasma	0.712	0.794	0.843	0.321
	sPLSDA	Plasma	0.717	0.796	0.859	0.366
Type III	PathCNN	Plasma	0.424	0.626	0.542	0.070
	eiCNN	Plasma	0.619	0.740	0.671	0.254
	MOGAT	Plasma	0.799	0.842	0.870	0.307
	eiNN	Plasma	0.821	0.859	0.910	0.393
	liNN	Plasma	0.772	0.821	0.886	0.395
	P-NET	Plasma	0.725	0.806	0.869	0.409
	MOGOnet	Plasma	0.840	0.873	0.872	0.412
	liCNN	Plasma	0.784	0.832	0.884	0.445
Pathformer	**Pathformer**	Plasma	**0.843**	**0.877**	**0.914**	**0.488**
Pathformer	**Pathformer**	Plasma (early-stage^b^)	0.853	0.869	0.916	0.479
Type I	eiLR	Platelet^c^	0.853	0.871	0.938	0.409
	eiRF	Platelet	0.826	0.853	0.930	0.418
	eiSVM	Platelet	0.752	0.802	0.886	0.423
	liSVM	Platelet	0.749	0.798	0.908	0.425
	liLR	Platelet	0.717	0.785	0.874	0.446
	liRF	Platelet	0.817	0.849	0.940	0.447
	liXGBoost	Platelet	0.879	0.897	**0.959**	0.453
	eiXGBoost	Platelet	0.853	0.875	0.939	0.461
Type II	sPLSDA	Platelet	0.711	0.767	0.849	0.253
	PLSDA	Platelet	0.788	0.826	0.899	0.370
Type III	PathCNN	Platelet	0.408	0.561	0.492	0.027
	eiCNN	Platelet	0.478	0.603	0.567	0.048
	liCNN	Platelet	0.579	0.671	0.662	0.134
	eiNN	Platelet	0.768	0.804	0.834	0.422
	P-NET	Platelet	0.548	0.659	0.934	0.439
	liNN	Platelet	0.843	0.873	0.909	0.445
	MOGAT	Platelet	0.702	0.772	0.923	0.445
	MOGOnet	Platelet	0.706	0.776	0.929	0.469
Pathformer	**Pathformer**	Platelet	**0.889**	**0.903**	0.938	**0.481**

a All cancer stages from I to IV.

b Cancer stage I and stage II.

c Stage information not available. All types of cancer patients are used as positives; the heathy controls are used as negatives. Each value is the mean of 5-fold cross-validation repeated twice (10 values).

The bold values are the highest in each dataset.

### 3.5 Biological interpretability of Pathformer in the data of cancer patient’s blood

**Figure 5.**
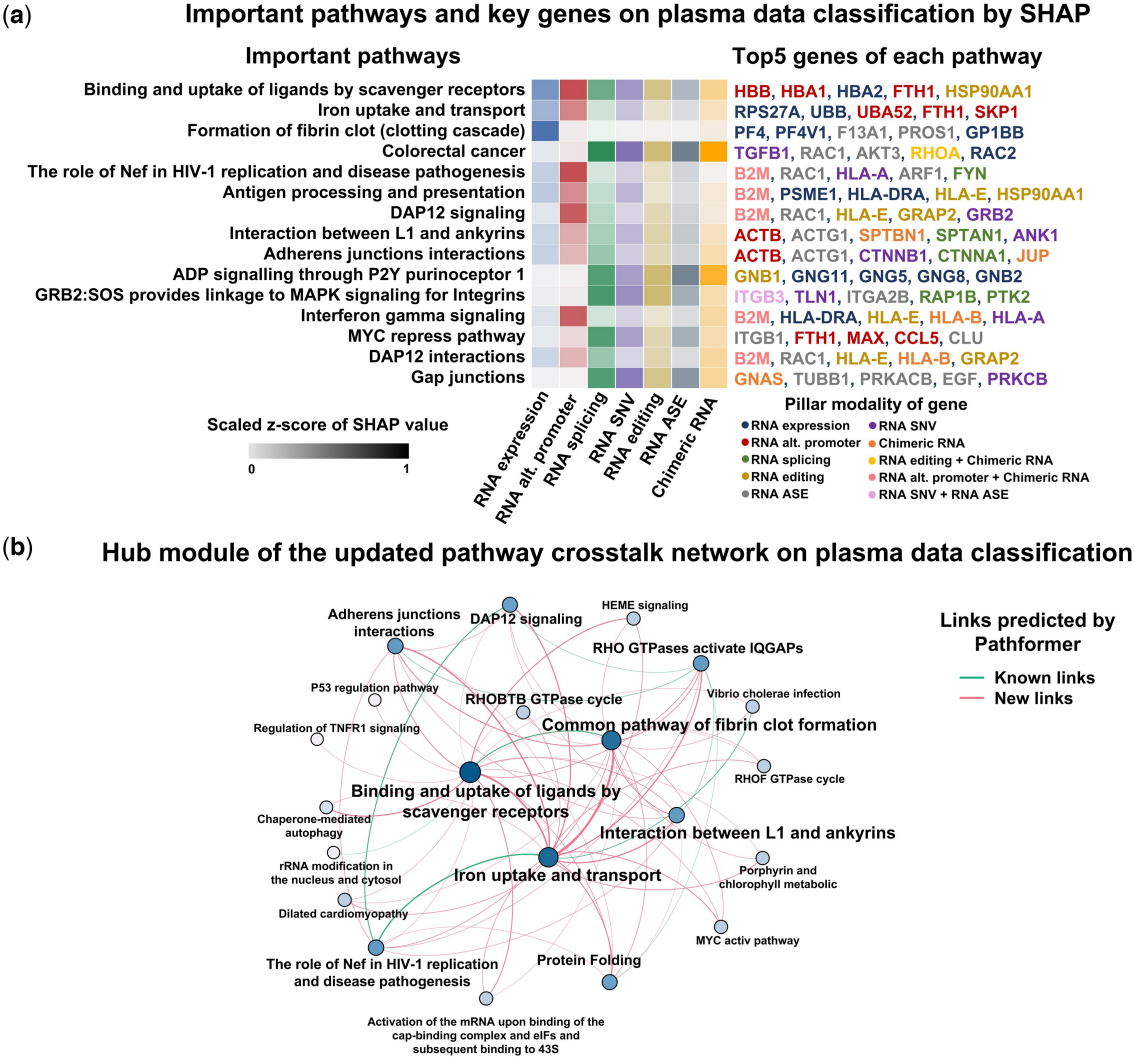
Biological interpretation of the cancer patients’ plasma data using Pathformer. (a) Important pathways and their key genes revealed by Pathformer in the plasma cell free RNA-seq data when classifying cancer patients from healthy controls. The pathways and their key genes were selected with top SHAP values. Among the key genes, different colors represent different pillar modalities (e.g. RNA expression, RNA editing, etc.) of the genes. (b) Hub modules of pathway crosstalk network are shown for plasma cell free RNA-seq data. Color depth and size of node represent the degree of node. Line thickness represents the weight of edge. All links are predicted by Pathformer, where known links are reported by the initial crosstalk network and new links are new predictions.

Based on the above analysis, we attempted to gain new insight into the deregulated alterations in plasma through Pathformer’s biological interpretability module (Fig. 5a and Supplementary Fig. S18a). First, we found that the pathways and genes ranked highly in SHAP values were associated with dysregulated alterations reported by previous experimental studies. For example, *binding and uptake of ligands* (e.g. oxidized low-density lipoprotein, oxLDL) *by scavenger receptors* pathway, with top SHAP value ranking, was reported to play a crucial role in cancer prognosis and carcinogenesis by promoting the degradation of harmful substances and accelerating the immune response (Ryu *et al.* 2020). Another two examples are *DAP12 signaling* pathway and *DAP12 interactions* pathway, which were highly ranked by SHAP value in both plasma and platelet data, were reported to regulate natural killer cell immune responses against certain tumor cells through platelet modulation cells (Campbell and Colonna 1999, Placke *et al.* 2011).

Furthermore, Pathformer can explore potential novel interactions between various biological processes in cancer patients’ plasma by updating pathway crosstalk network (Fig. 5b). For example, the link between *binding and uptake of ligands by scavenger receptors* pathway and *iron uptake and transport* pathway was a novel addition to the known links (Pathformer’s input pathway crosstalk network curated from published databases, see Methods). This finding aligns with a previous report of SCARA5 (scavenger receptor class A member) as a ferritin receptor (Yu *et al.* 2020). The crosstalk between two pathways was amplified by Pathformer in plasma dataset, probably because they were important for classification. In summary, Pathformer’s updated pathway crosstalk network can effectively visualize the information flow between pathways related to cancer classification tasks, providing new insight into the crosstalk of biological pathways in cancer patients’ plasma.

## 4 Conclusion and discussion

Pathformer successfully applied a Transformer model to integrate multi-modal data for cancer diagnosis and prognosis. Particularly, it introduced a novel biological embedding method based on the compacted multi-modal vectors (Fig. 1b). Moreover, it utilized the criss-cross attention mechanism of Transformer to capture crosstalk between biological pathways and regulation between modalities (i.e. different omics).

### 4.1 Clinical applications of Pathformer

Pathformer can be applied to various classification tasks in disease diagnosis, treatment, and prognosis, such as early detection, cancer staging, survival prediction, and drug response prediction. Its predictive accuracy, stability, reliability, and biological interpretability were demonstrated through substantial benchmark and case studies focusing on cancer prognosis and noninvasive diagnosis. Ablation analysis demonstrated the pivotal role of various modal integrations and core modules (CC-attention, PNSS, and pathway embedding) in Pathformer for accurate classification. Our discussion on explained variances across integration models and multi-omics data further corroborated conclusions from benchmark tests and ablation analyses (Supplementary Note S11). Moreover, this framework is adaptable for the diagnosis and prognosis of other complex diseases, like autoimmune disease, neurodegenerative diseases, etc.

### 4.2 Potential targets revealed in cancer patients’ blood

Particularly, we identified some potential noninvasive cancer diagnostic biomarkers by Pathformer, such as the scavenger receptor related pathways and DAP12 related pathways, which are associated with extracellular vesicle transport (Kzhyshkowska *et al.* 2006) and immune response (Campbell and Colonna 1999), respectively. We even found a new cancer-related pathway crosstalk in blood, which is between *binding and uptake of ligands by scavenger receptors* pathway and *iron uptake and transport* pathway. These results provide candidate targets for the mechanism study of cancer microenvironment and immune system, and even new targets for cancer treatment.

### 4.3 Limitations of Pathformer and future directions

For gene selection, Pathformer used genes involved in four common pathway databases, all of which consist of protein-coding genes. However, a substantial body of literature has reported that noncoding RNAs are also crucial in cancer prognosis and diagnosis (Qi *et al.* 2016). Therefore, incorporating noncoding RNAs and their related functional pathways into Pathformer would be promising for future work. For clinical applications in liquid biopsy, we used the multi-modal features derived from cfRNA-seq only in the application of liquid biopsy, because the published cell-free multi-omics datasets (Tao *et al.* 2023) are usually too small to be train-and-tested. For computational efficiency and memory costs, there is still room for improvement for Pathformer. Pathway embedding of Pathformer has prevented memory overflow of Transformer module caused by long inputs, but training still requires significant time and space (Supplementary Note S12). Therefore, when adding more pathways or gene sets (e.g. transcription factors), Pathformer still faces the issue of memory overflow. In the future work, we may introduce linear attention to further improve computational speed. Furthermore, potential signatures, regulations and biomarkers identified by Pathformer are also needed to be studied and validated by further biological experiments and clinical tests.

## Supplementary Material

btae316_Supplementary_Data

## Data Availability

All datasets used in this study are publicly available for academic research usages. The TCGA datasets were derived from sources in the public domain: https://www.cancer.gov/ccg/. The plasma dataset is available in Gene Expression Omnibus (https://www.ncbi.nlm.nih.gov/geo/) under accession codes GSE174302 and GSE186607. The platelet dataset is available in Gene Expression Omnibus (https://www.ncbi.nlm.nih.gov/geo/) under accession codes GSE68086 and GSE89843. The details of usage are also fully illustrated in Methods and Supplementary Notes. Source code for data preprocessing and model training is freely available at Github (https://github.com/lulab/Pathformer) with detailed instructions. Source code for comparing the other methods is also included.
