# Supplementary material for: Pathformer: a biological pathway informed transformer for disease diagnosis and prognosis using multi-omics data
Source: Bioinformatics. 2024 May 13;40(5):btae316. doi: 10.1093/bioinformatics/btae316 (PMC11139513; doi:10.1093/bioinformatics/btae316)
Supplement: btae316_Supplementary_Data [file btae316_supplementary_data.zip › Supplementary Figures and Notes.doc]

**Supplementary information**

[Supplementary Figures 3](#_Toc162445801)

[Supplementary figure 1: A block of Transformer module with pathway crosstalk network bias. 3](#_Toc162445802)

[Supplementary figure 2: Benchmark datasets. 4](#_Toc162445803)

[Supplementary figure 3: Optimal combination of hyperparameters. 5](#_Toc162445804)

[Supplementary figure 4: Convergence analysis of Pathformer. 6](#_Toc162445805)

[Supplementary figure 5: Confidence score histograms and calibration curves of Pathformer. 7](#_Toc162445806)

[Supplementary figure 6: Various accuracy metrics under different thresholds of Pathformer’s confidence scores. 8](#_Toc162445807)

[Supplementary figure 7: Correlations between confidence scores and prediction accuracy for various multi-modal integration methods. 9](#_Toc162445808)

[Supplementary figure 8: Ablation analysis of Pathformer for the classification of early- and late-stage cancer patients. 10](#_Toc162445809)

[Supplementary figure 9: Ablation analysis of Pathformer for drug response prediction. 11](#_Toc162445810)

[Supplementary figure 10: BRCA early- and late- stage classification related modalities, pathways and genes revealed by Pathformer. 12](#_Toc162445811)

[Supplementary figure 11: Keplan-Meier curves of active pathway selected identified by Pathformer on BRCA survival risk classification. 13](#_Toc162445812)

[Supplementary figure 12: Decision process analysis of Pathformer on BRCA survival risk classification. 14](#_Toc162445813)

[Supplementary figure 13: Decision process analysis of Pathformer on BRCA early- and late- stage classification. 15](#_Toc162445814)

[Supplementary figure 14: Commonality analysis of Pathformer’s decision process on BRCA survival risk classification and stage classification. 16](#_Toc162445815)

[Supplementary figure 15: Uncovering new patterns in TCGA pan-cancer data using key features identified by Pathformer. 17](#_Toc162445816)

[Supplementary figure 16: Performances of Pathformer with different modalities on liquid biopsy datasets. 18](#_Toc162445817)

[Supplementary figure 17: Pathformer integrates multi-modal liquid biopsy data for noninvasive cancer diagnosis. 19](#_Toc162445818)

[Supplementary figure 18: Interpretation of cancer patients’ platelet data using Pathformer. 20](#_Toc162445819)

[Supplementary figure 19: Decision process analysis of Pathformer on plasma data. 21](#_Toc162445820)

[Supplementary figure 20: Decision process analysis of Pathformer on platelet data. 22](#_Toc162445821)

[Supplementary figure 21: Explained variances of different models and modalities. 23](#_Toc162445822)

[Supplementary Notes 24](#_Toc162445823)

[Supplementary Note 1: Pathway crosstalk network calculation 24](#_Toc162445824)

[Supplementary Note 2: Multi-omics data collection and preprocessing 24](#_Toc162445825)

[Supplementary Note 3: Conversion function in gene embedding 25](#_Toc162445828)

[Supplementary Note 4: Classification calculation of Pathformer 25](#_Toc162445829)

[Supplementary Note 5: Biological interpretability module 26](#_Toc162445830)

[Supplementary Note 6: Details of model training and test 27](#_Toc162445831)

[Supplementary Note 7: Comparison methods 28](#_Toc162445832)

[Supplementary Note 8: Reliability analysis of Pathformer’s confidence scores 30](#_Toc162445841)

[Supplementary Note 9: Correct predictions analysis 30](#_Toc162445842)

[Supplementary Note 10: Feature selection for cancer noninvasive diagnosis 31](#_Toc162445843)

[Supplementary Note 11: Explained variances of different models and modalities 31](#_Toc162445844)

[Supplementary Note 12: Pathformer’s resource and time consumption 32](#_Toc162445845)

[References 33](#_Toc162445846)

# Supplementary Figures

## Supplementary figure 1: A block of Transformer module with pathway crosstalk network bias.

The pathway embedding matrix is used as input and the pathway crosstalk network matrix is used as bias. $N_{p}$, number of pathways; $D_{p}$, dimensionality of pathway embedding; *h*, number of attention heads; *d*, attention dimension; $\boldsymbol{V}_{1},$ $\boldsymbol{K}_{1}$, $\boldsymbol{Q}_{1}$, $\boldsymbol{A}_{1}:$ value, key, query and attention map of col-attention; $\boldsymbol{V}_{2}$, $\boldsymbol{K}_{2}$, $\boldsymbol{Q}_{2}$, $\boldsymbol{A}_{2}:$ value, key, query and attention map of row-attention; $+$, element-wise addition; $\times$, matrix multiplication; $\circ$, matrix dot product; $\beta$, constant coefficient for row-attention.

## Supplementary figure 2: Benchmark datasets.

We collected cancer datasets from TCGA covering three types of tasks: cancer high- and low-risk survival classification, cancer early- and late-stage classification, and clinical drug response prediction. For cancer low- and high- survival risk classification, we defined samples with survival time greater than 1825 days as low-risk samaples and those less than 1825 days as high-risk samples. Pan-cancer dataset of survival classification contains 33 cancer types of TCGA terms. For cancer early- and late- stage classification, we defined stage I and stage II as the early-stage and stage III as the late-stage. Pan-cancer dataset of stage classification contains 21 cancer types of TCGA terms. For clinical drug response prediction, we defined samples with complete response and partial response as responders, and samples with stable disease and progressive disease as non-responders. Each dataset name is an abbreviation of cancer type according to the TCGA terms. More details are in **Supplementary Note 2**.

## Supplementary figure 3: Optimal combination of hyperparameters.

Optimal combination of hyperparameters in one experiment for (**a**) BRCA early- and late- stage classification and (**b**) BRCA high- and low- risk survival classification, demonstrating how to optimize parameters. First, we gave some hyperparameters of Pathformer based on prior experience, such as the number of blocks and the number of multi-head self-attention. Subsequently, we aimed to determine the optimal value of 8 combinations consisting of maximum of learning rate, dropout probability of classification (*c*), and constant coefficient for row-attention ($\beta$). Then, we performed 5-fold cross-validation on the training set for hyperparameter optimization. We used the F1score_macro as the selection criterion and used grid search to find the optimal combination of hyperparameters. More details are in **Supplementary Note 6** and **Supplementary Table 2**.

## Supplementary figure 4: Convergence analysis of Pathformer.

We depicted the training loss in relation to the number of epochs on multiple TCGA datasets and the liquid biopsy datasets, verifying the convergence of Pathformer. The total loss for each dataset decreases rapidly as the iterations progress, ultimately converging to a stable state. Furthermore, the specific number of training epochs for Pathformer on each dataset is determined by the stopping criteria of the early stopping strategy (for more details, refer to **Supplementary Note 6**).

## Supplementary figure 5: Confidence score histograms and calibration curves of Pathformer.

We depicted the confidence score histograms and calibration curves of Pathformer on multiple TCGA datasets and the liquid biopsy datasets, verifying the reliability of Pathformer. We binned confidence scores from 0 to 1 into 10 intervals with width 0.1 each, and counted the number of samples and the frequency of the positive label in each interval. Across various datasets, Pathformer’s fraction of positives generally correlate positively with confidence scores, although not linearly. The confidence score histograms show balance property in most datasets, akin to logistic regression, likely due to our model’s utilization of the softmax function to transform confidence scores. More details are in **Supplementary Note 8**.

## Supplementary figure 6: Various accuracy metrics under different thresholds of Pathformer’s confidence scores.

We depicted the relationship between confidence scores and various accuracy metrics (accuracy, F1 score, precision, and recall) of Pathformer on multiple TCGA datasets and the liquid biopsy datasets. Similar to calibration curves, we select confidence scores at intervals of 0.1 as thresholds, predicting samples below these thresholds as negative and above as positive, to calculate the accuracy, F1score_macro, precision and recall at each threshold. We observed that as the threshold increases, precision continually rises, recall gradually decreases, accuracy initially increases and then stabilizes, and the F1 score captures the equilibrium between precision and recall. This indicates the rationality of Pathformer's confidence scores.

## Supplementary figure 7: Correlations between confidence scores and prediction accuracy for various multi-modal integration methods.

Each box plot represents the pearson correlation coefficients between confidence scores and fraction of positives on test sets of all datasets for different integration models. Error bars are from 5-fold cross-validation repeated twice (10 values) of all datasets. More details are in **Supplementary Note 8**.

## Supplementary figure 8: Ablation analysis of Pathformer for the classification of early- and late-stage cancer patients.

**a**. Different types of input modalities (omics data types) were used as input for TCGA cancer early- and late-stage classification. **b**. Ablation analysis of different calculation modules in Pathformer. Error bars are from 2 times 5-fold cross-validation across 9 datasets, representing 95% confidence intervals. CC-attention, Pathformer without pathway crosstalk network bias; Transformer, Pathformer with only normal attention and pathway embedding; PSNN, Pathformer with only classification module with pathway embedding; NN, Pathformer with only classification module with gene embedding.

## Supplementary figure 9: Ablation analysis of Pathformer for drug response prediction.

**a**. Different types of input modalities (omics data types) were used as input for 5 TCGA datasets of drug response prediction. **b**. Ablation analysis of different calculation modules in Pathformer. Error bars are from 2 times 5-fold cross-validation across 5 datasets, representing 95% confidence intervals. CC-attention, Pathformer without pathway crosstalk network bias; Transformer, Pathformer with only normal attention and pathway embedding; PSNN, Pathformer with only classification module with pathway embedding; NN, Pathformer with only classification module with gene embedding.

## Supplementary figure 10: BRCA early- and late- stage classification related modalities, pathways and genes revealed by Pathformer.

**a**. Contributions of different modalities for BRCA early- and late- stage classification calculated by attention weights. **b**. Important pathways and their key genes with top SHapley Additive exPlanations (SHAP) values. Among the key genes, different colors represent different pillar modalities of the genes. **c**. A hub module of the updated pathway crosstalk network for BRCA early- and late- stage classification. Color depth and size of node represents the degree of node. Line thickness represents the weight of edge. All links are predicted by Pathformer, where known links are reported by the initial crosstalk network and new links are new predictions. In breast cancer early- and late-stage classification, *iron uptake and transport* pathway had the greatest impact. Supportively, the transport and storage of iron in cells are known to play a key role in carcinogenesis, cell proliferation, and the development of breast cancer(Marques, et al., 2014).

## Supplementary figure 11: Keplan-Meier curves of active pathway selected identified by Pathformer on BRCA survival risk classification.

Keplan-Meier curves is depicted the hierarchical relationship between patients with high and low scores in active pathways. The pathway score for each sample was obtained by averaging across different dimensions of pathway embedding. P-value calculated through Log-Rank test.

## Supplementary figure 12: Decision process analysis of Pathformer on BRCA survival risk classification.

**a**. Principal component analysis (PCA) of pathway embedding matrices before and after Pathformer update, demonstrating the feature extraction capability of Pathformer visually. **b.** Heatmap of pathway scores updated by Pathformer for the top 15 pathways ranked by importance on BRCA survival risk classification. Pathway score for each sample was obtained by averaging across different dimensions of pathway embedding updated by Pathformer. Proportion of BRCA subtype (**c**) and age (**d**) among samples correctly and incorrectly predicted by Pathformer on BRCA survival risk classification. P-value calculated through chi-square test.

## Supplementary figure 13: Decision process analysis of Pathformer on BRCA early- and late- stage classification.

**a**. Principal component analysis (PCA) of pathway embedding matrices before and after Pathformer update, demonstrating the feature extraction capability of Pathformer visually. **b.** Heatmap of pathway scores updated by Pathformer for the top 15 pathways ranked by importance on BRCA early- and late- stage classification. Pathway score for each sample was obtained by averaging across different dimensions of pathway embedding updated by Pathformer. Proportion of BRCA subtypes (**c**) and age (**d**) among samples correctly and incorrectly predicted by Pathformer on BRCA early- and late- stage classification. P-value calculated through chi-square test.

## Supplementary figure 14: Commonality analysis of Pathformer’s decision process on BRCA survival risk classification and stage classification.

**a**. Scatter plot of the importance of pathways on breast cancer survival risk and stage classification. Red points indicate pathways highly important in both classification tasks (SHAP value > 0.06). **b.** Proportion of BRCA subtypes (**c**) and age (**d**) among samples accurately predicted by Pathformer on both tasks and other samples. P-value calculated through chi-square test.

## Supplementary figure 15: Uncovering new patterns in TCGA pan-cancer data using key features identified by Pathformer.

**a**. Scatter plot of the importance of pathways on pan-cancer survival risk and stage classification. Red points indicate pathways highly important in both classification tasks (SHAP value > 0.04). **b**. tSNE representation of initial pathway embedding matrix before Pathformer update. Here, initial pathway embedding matrix is about 45 active pathways from (**a**). Each point’s color represents the corresponding sample’s cancer type. **c**. tSNE representation of pathway embedding matrices updated by Pathformer for pan-cancer survival risk and stage classification, respectively. Here, pathway embedding matrices updated by Pathformer are about 45 active pathways from (**a**). Each point’s color represents the corresponding sample’s prediction result, cancer type, age, and gender.

## Supplementary figure 16: Performances of Pathformer with different modalities on liquid biopsy datasets.

Error bars obtained from 2 times 5-fold cross-validation and represent 95% confidence intervals of various evaluation scores.

## Supplementary figure 17: Pathformer integrates multi-modal liquid biopsy data for noninvasive cancer diagnosis.

**a**. Contributions of different input features and their statistical indicators when classifying cancer patients from healthy controls by seven RNA-level modalities on two liquid biopsy datasets (cell free RNA-seq). All mean represents the sum of mean, weighted mean and window weighted mean. Each type of RNA splicing is the sum of all statistical indicators in this type. **b**. Classification performance of different input combinations on two liquid biopsy datasets. Each value is the mean of 2 times 5-fold cross-validation. Top 1, Top 3, and Top 5 respectively represent the top-contributing modality combinations, the top three-contributing modality combinations, and the top five-contributing modality combinations, evaluated by Pathformer on each dataset. For example, in plasma dataset, the top three-contributing modality combinations are RNA expression, RNA alternative promoter, and RNA single nucleotide variations. In platelet dataset, the top three-contributing modality combinations are RNA expression, RNA alternative promoter, and RNA splicing.

## Supplementary figure 18: Interpretation of cancer patients’ platelet data using Pathformer.

**a**. Important pathways and their key genes revealed by Pathformer in the platelet cell free RNA-seq data when classifying cancer patients from healthy controls. The pathways and their key genes were selected with top SHAP values. Among the key genes, different colors represent different pillar modalities of the genes. **b**. Hub modules of pathway crosstalk network are shown for platelet cell free RNA-seq data. Color depth and size of node represent the degree of node. Line thickness represents the weight of edge. All links are predicted by Pathformer, where known links are reported by the initial crosstalk network and new links are new predictions.

## Supplementary figure 19: Decision process analysis of Pathformer on plasma data.

**a**. Principal component analysis (PCA) of pathway embedding matrices before and after Pathformer update, demonstrating the feature extraction capability of Pathformer visually. **b.** Heatmap of pathway scores updated by Pathformer for the top 15 pathways ranked by importance on plasma cell free RNA-seq data. Pathway score for each sample was obtained by averaging across different dimensions of pathway embedding updated by Pathformer. Proportion of gender (**c**), age (**d**), and cancer type (**e**) among samples correctly and incorrectly predicted by Pathformer on plasma cell free RNA-seq data. P-value calculated through chi-square test.

## Supplementary figure 20: Decision process analysis of Pathformer on platelet data.

**a**. Principal component analysis (PCA) of pathway embedding matrices before and after Pathformer update, demonstrating the feature extraction capability of Pathformer visually. **b.** Heatmap of pathway scores updated by Pathformer for the top 15 pathways ranked by importance on platelet cell free RNA-seq data. Pathway score for each sample was obtained by averaging across different dimensions of pathway embedding updated by Pathformer. Proportion of gender (**c**), age (**d**), and cancer type (**e**) among samples correctly and incorrectly predicted by Pathformer on platelet cell free RNA-seq data. P-value calculated through chi-square test.

## Supplementary figure 21: Explained variances of different models and modalities.

The explained variances of Pathformer and other 18 integration models are depicted on (**a**) TCGA datasets for cancer survival risk classification, stage classification, and drug response prediction tasks, and (**b**) liquid biopsy datasets for cancer diagnosis. The explained variances of Pathformer with different modalities as inputs are depicted on (**a**) TCGA datasets and (**b**) liquid biopsy datasets. Error bars are from 5-fold cross-validation repeated twice (10 values) of all datasets. More details are in **Supplementary Note 11**.

# Supplementary Notes

## Supplementary Note 1: Pathway crosstalk network calculation

We used *BinoX*, a classic tool for crosstalk analysis, to calculate the crosstalk relationship of 1,497 pathways and form a pathway crosstalk network. *BinoX* uses the relationship between genes of two pathways in genome-wide functional association networks to calculate the degree of association between two pathways, that is, the crosstalk between two pathways. We used FunCoup v3.0 database(Schmitt, et al., 2014) to obtain genome-wide functional association networks. Then, we set the cut off for the link weight to 0.75, the number of iterations for the sampling method to 100, and the minimum number of nodes per group to 15 on *BinoX* software.

## Supplementary Note 2: Multi-omics data collection and preprocessing

### *TCGA data*

For benchmark testing, we collected TCGA datasets to evaluate classification performance of Pathformer and existing comparison methods in two classification tasks, including cancer early- and late- stage classification, cancer low- and high- survival risk classification, and clinical drug response prediction (**Supplementary Fig. 2**).

Firstly, we used the “TCGAbiolinks” package of *R* software to download RNA expression, DNA methylation, DNA CNV and clinical data of TCGA datasets. Among these downloaded datasets, the RNA expression values were read counts processed by *STAR* and normalized by TPM, the CpG site levels of DNA methylation data are β-values measured using the Infinium HumanMethylation450 BeadChip, and the DNA CNV data were masked copy number segment and gene level score processed by *Gistic2*.

Next, we added label information for classification experiments. For cancer early- and late- stage classification, we defined stage I and stage II as the early-stage and stage III as the late-stage according to the "pathologic stage" information in clinical data. For cancer low- and high- survival risk classification, we defined samples from patients with survival time greater than 1825 days as low-risk samples and those less than 1825 days as high-risk samples. For clinical drug response prediction, we defined samples with complete response and partial response as responders, and samples with stable disease and progressive disease as non-responders(Chiu, et al., 2019; Sharifi-Noghabi, et al., 2019), based on the clinical annotations from Ding et al.’s supplementary materials(Ding, et al., 2016).

Finally, we conducted additional filtering. Firstly, we retained only those samples in each cancer dataset that included RNA expression, DNA methylation, DNA CNV, and their corresponding clinical labels. Specifically, for clinical drug response prediction, we filtered out samples with inconsistent responses to a particular drug. Secondly, we specifically selected cancer datasets/drug datasets with a large number of samples, including those for stage classification with over 300 samples, those for survival classification with over 150 samples, and those for drug response prediction with over 100 samples.

Specifically, cancer low- and high- survival risk classification task involves 10 datasets from TCGA term, including breast cancer (BRCA), neck cancer (HNSC), low-grade gliomas (LGG), bladder cancer (BLCA), melanoma (SKCM), kidney clear cell carcinoma (KIRC), lung adenocarcinoma (LUAD), lung squamous cell carcinoma (LUSC), liver cancer (LIHC) and pan-cancer. The pan-cancer dataset contains 3447 samples, covering 33 cancer types (BRCA KIRC, HNSC, LGG, LUAD, LUSC, STAD, BLCA, LIHC, SKCM, THCA, KICH, COAD, KIRP, READ, UCEC, ACC, CESC, CHOL, DLBC, ESCA, GBM, LAML, MESO, OV, PAAD, PCPG, PRAD, SARC, TGCT, THYM, UCS, UVM, abbreviation of cancer type according to the TCGA terms). Cancer early- and late- stage classification task involves 10 datasets from TCGA, including BRCA, BLCA, SKCM, stomach cancer (STAD), KIRC, LUAD, LUSC, LIHC, thyroid cancer (THCA) and pan-cancer. The pan-cancer dataset contains 5610 samples, covering 21 cancer types (BRCA, COAD, HNSC, KICH, KIRC, KIRP, LUAD, LUSC, READ, STAD, ACC, BLCA, CHOL, ESCA, LIHC, MESO, PAAD, SKCM, TGCT, THCA, UVM, abbreviation of cancer type according to the TCGA terms). Clinical drug response prediction task involves five drug datasets from TCGA, including Cisplatin, Carboplatin, Gemcitabine, Paclitaxel, and 5-Fluorouracil. Each drug dataset contains samples from various cancer types.

### *Liquid biopsy data*

To further verify the effectiveness of Pathformer in cancer diagnosis, we collected two complex body fluid datasets from different blood components: the plasma dataset (comprising 98 healthy donors, 90 colorectal cancer (CRC), 23 esophageal cancer (ESCA), 71 STAD, 57 LIHC, and 34 LUAD assayed by total cell-free RNA-seq(Chen, et al., 2022; Tao, et al., 2023) and the platelet dataset (comprising 286 healthy donors, 462 LUAD, 42 CRC, 40 GBM, 39 BRCA, 35 PAAD, and 14 LIHC from two studies assayed by tumor-educated blood platelet RNA-seq(Best, et al., 2017; Best, et al., 2015)). For body fluid datasets, we used seven modalities at the RNA level as Pathformer’s input, including RNA expression, RNA splicing, RNA editing, RNA alternative promoter (RNA alt. promoter), RNA allele-specific expression (RNA ASE), RNA single nucleotide variations (RNA SNV), and chimeric RNA.

We used a bioinformatics pipeline to preprocess raw sequence reads into datasets of different modalities. Firstly, we used *cutadapt* tool to trim adaptors and low-quality reads, and then removed the reads which can be mapped to ERCC’s spike-in sequences, NCBI’s UniVec sequences (vector contamination), and human rRNA sequences by *STAR* software. Next, we applied STAR software to map all the retained unmapped reads to the hg38(Schneider, et al., 2017) genome index built with the GENCODE v27(Harrow, et al., 2012) annotation and calculated seven modalities at the RNA level based on the mapping result. The details of the calculation processes are as follows: (1) RNA expression data were read counts aggerated to gene by *featureCounts* and were normalized by TPM. (2) RNA alternative promoter data represented transcript isoform abundances quantified by salmon and were normalized by TPM. We only selected isoforms with transcription start sites within 10 bp (sharing the same promoter) and TPMs greater than 1(6 7 Lehmann Kjong-Van 3 4 6 7 Liu Fenglin 10 Shiraishi Yuichi 11 Soulette Cameron M. 12 Urban Lara 2, et al., 2020). (3) RNA splicing data were a series of alternative splicing events with the percent spliced-in (PSI) score calculated using *rMATs-turbo*. (4) As for RNA editing data, editing sites were identified by *GATK ASEReadCounter* based on REDIportal(Picardi, et al., 2017) and editing ratios of editing sites were defined as allele count divided by total count. (5) In RNA allele-specific expression data, allele-specific expression gene site were identified by *GATK ASEReadCounter* based on SNP sites and allelic expressions (AE, AE = |0.5 − Reference ratio|, ﻿Reference ratio = Reference reads/Total reads) were calculated for all sites with ≥16 reads(Castel, et al., 2015). (6) In RNA single nucleotide variations data, *GATK SplitNCigarReads* was used to split intron-spanning reads for confident SNP calling at RNA level. *GATK HaplotypeCaller* and *GATK VariantFilteration* were used to identify and filter alterations. Allele fraction was defined as allele count divided by total count (reference count and allele count). (7) Chimeric RNA data were identified by remapping unaligned reads to chimeric junctions by *STAR-fusion*. Chimera references were based on GTex(Singh, et al., 2020) and ChimerDB-v3(Lee, et al., 2017).

## Supplementary Note 3: Conversion function in gene embedding

In gene embedding, we use the conversion function $\boldsymbol{F}_{E}$, a series of statistical indicator functions, to uniformly convert different modalities into gene level modal features. These statistical indicator functions include gene level score ($f_{1}$), count ($f_{2}$), minimum ($f_{3}$), maximum ($f_{4}$), mean ($f_{5}$), entropy ($f_{6}$), weighted mean in whole gene ($f_{7}$) and weighted mean in window ($f_{8}$). The formulas are as follows:

$$f_{1}\left( \boldsymbol{M}_{i} \right)=\left[ X_{g_{1}}^{(i)},\cdots,X_{g_{N_{g}}}^{(i)} \right]\boldsymbol{\in}\mathbb{R}^{N_{g}}$$

$$f_{2}\left( \boldsymbol{M}_{i} \right)=[count\left( \boldsymbol{X}_{{ge}_{1}}^{\boldsymbol{(}i\boldsymbol{)}} \right),\cdots,count(\boldsymbol{X}_{{ge}_{N_{g}}}^{\boldsymbol{(}i\boldsymbol{)}})]\boldsymbol{\in}\mathbb{R}^{N_{g}}$$

$$f_{3}(\boldsymbol{M}_{i})=[min\left( \boldsymbol{X}_{{ge}_{1}}^{\boldsymbol{(}i\boldsymbol{)}} \right),\cdots,min(\boldsymbol{X}_{{ge}_{N_{g}}}^{\boldsymbol{(}i\boldsymbol{)}})]\boldsymbol{\in}\mathbb{R}^{N_{g}}$$

$$f_{4}(\boldsymbol{M}_{i})=[max\left( \boldsymbol{X}_{{ge}_{1}}^{\boldsymbol{(}i\boldsymbol{)}} \right),\cdots,max(\boldsymbol{X}_{{ge}_{N_{g}}}^{\boldsymbol{(}i\boldsymbol{)}})]\boldsymbol{\in}\mathbb{R}^{N_{g}}$$

$$f_{5}(\boldsymbol{M}_{i})=[mean\left( \boldsymbol{X}_{{ge}_{1}}^{\boldsymbol{(}i\boldsymbol{)}} \right),\cdots,mean(\boldsymbol{X}_{{ge}_{N_{g}}}^{\boldsymbol{(}i\boldsymbol{)}})]\boldsymbol{\in}\mathbb{R}^{N_{g}}$$

$$f_{6}\left( \boldsymbol{M}_{i} \right)=[entropy\left( \boldsymbol{X}_{{ge}_{1}}^{\boldsymbol{(}i\boldsymbol{)}} \right),\cdots,entropy(\boldsymbol{X}_{{ge}_{N_{g}}}^{\boldsymbol{(}i\boldsymbol{)}})]\boldsymbol{\in}\mathbb{R}^{N_{g}}$$

$$f_{7}\left( \boldsymbol{M}_{i} \right)=[weighted\_mean\left( \boldsymbol{X}_{{ge}_{1}}^{\boldsymbol{(}i\boldsymbol{)}} \right),\cdots,weighted\_mean(\boldsymbol{X}_{{ge}_{N_{g}}}^{\boldsymbol{(}i\boldsymbol{)}})]\boldsymbol{\in}\mathbb{R}^{N_{g}}$$

$$weighted\_mean(\boldsymbol{X}_{{ge}_{l}}^{\left( i \right)})=({mc}_{1}^{\left( i \right)\left( l \right)}+\cdots+{mc}_{t_{l}}^{\left( i \right)\left( l \right)})/({tc}_{1}^{\left( i \right)\left( l \right)}+\cdots+{tc}_{t_{l}}^{\left( i \right)\left( l \right)}), x_{t}^{\left( i \right)\left( l \right)}={mc}_{t}^{\left( i \right)\left( l \right)}/{tc}_{t}^{\left( i \right)\left( l \right)}$$

$$f_{8}\left( \boldsymbol{M}_{i} \right)=\left[ \left[ f_{7}\left( \boldsymbol{M}_{i}^{window1} \right),f_{7}\left( \boldsymbol{M}_{i}^{window2} \right),f_{7}\left( \boldsymbol{M}_{i}^{window3} \right) \right] \right]\boldsymbol{\in}\mathbb{R}^{N_{g}\times3}$$

, where $X_{g_{l}}^{(i)}$ is the score of the *i*th modality corresponding to the *l*th gene, $\boldsymbol{X}_{{ge}_{l}}^{\boldsymbol{(}i\boldsymbol{)}}=\left[ x_{1}^{(i)(l)},x_{2}^{(i)(l)}, \cdots,x_{t_{l}}^{(i)(l)} \right]$ is the event vector mapped to the *l*th gene in the *i*th modality;$t_{l}$ is the number of events mapped to the *l*th gene; $x_{t}^{(i)(l)}$ is the score of the *t*th event of the *l*th gene in the *i*th modality; ${mc}_{t}^{\left( i \right)\left( l \right)}$, ${tc}_{t}^{\left( i \right)\left( l \right)}$are the number of mutated reads and total reads of the *t*th event of the *l*th gene in the *i*th modality; $\boldsymbol{M}_{i}^{window1}=\left[ X_{g_{1}}^{(i)(window1)},\cdots,X_{g_{N_{g}}}^{(i)(window1)} \right]$, and $X_{g_{l}}^{(i)(window1)}$ is 1/3 event vector mapped to the *l*th gene in the *i*th modality. Conversion function of modality *i* is constructed from distinct statistical indicator functions (more details in **Supplementary Table 1**). When the length of gene embedding $D_{g}$ is still less than 2 after processing by conversion functions $\boldsymbol{F}_{E}$, we use a fully connected neural network layer to transform gene embedding to 32 dimensions.

## Supplementary Note 4: Classification calculation of Pathformer

Pathformer uses the Transformer module based on criss-cross attention with pathway crosstalk network bias, which has 3 blocks. We used superscripts with parenthesis to represent data at different layers, where $\boldsymbol{E}_{P}^{(0)}=\boldsymbol{E}_{P}$, $\boldsymbol{P}^{(0)}=\boldsymbol{P}$ is the data before entering the first layer, $\boldsymbol{E}_{P}$ is initial pathway embedding, and $\boldsymbol{P}$ is initial pathway crosstalk network matrix. Pathformer is calculated as follows:

$$\boldsymbol{E}_{P}^{(1)}, \boldsymbol{P}^{(1)}=\mathrm{Transformer}(\boldsymbol{E}_{P}^{(0)}, \boldsymbol{P}^{(0)})$$

$$\boldsymbol{E}_{P}^{(2)}, \boldsymbol{P}^{(2)}\boldsymbol{=}\mathrm{Transformer}\boldsymbol{(}\boldsymbol{E}_{P}^{(1)}, \boldsymbol{P}^{(1)}\boldsymbol{)}$$

$$\boldsymbol{E}_{P}^{(3)}, \boldsymbol{P}^{(3)}\boldsymbol{=}\mathrm{Transformer}\boldsymbol{(}\boldsymbol{E}_{P}^{(2)}, \boldsymbol{P}^{(2)}\boldsymbol{)}$$

In order to solve classification tasks, we used a fully connected neural network as the classification module to transform pathway embedding encoded by the Transformer module into the probability for each label. The calculation is as follows:

$$\boldsymbol{L}_{1}= \mathrm{Flatten}(\boldsymbol{E}_{P}^{(3)})$$

$$\boldsymbol{Z}_{1}= \mathrm{dropout}_{c}(\mathrm{RELU}(\boldsymbol{L}_{1}\boldsymbol{W}_{c1}+\boldsymbol{B}_{c1}))$$

$$\boldsymbol{Z}_{2}= \mathrm{dropout}_{c}(\mathrm{RELU}(\boldsymbol{Z}_{1}\boldsymbol{W}_{c2}+\boldsymbol{B}_{c2}))$$

$$\boldsymbol{Z}_{3}= \mathrm{dropout}_{c}(\mathrm{RELU}(\boldsymbol{Z}_{2}\boldsymbol{W}_{c3}+\boldsymbol{B}_{c3}))$$

$$\hat{Y}=softmax(\boldsymbol{Z}_{3}\boldsymbol{W}_{out}+\boldsymbol{B}_{out})$$

, where $\boldsymbol{W}_{c1}\boldsymbol{\in}\mathbb{R}^{{(N}_{p}*D_{p})\times d_{c1}}$, $\boldsymbol{W}_{c2}\boldsymbol{\in}\mathbb{R}^{d_{c1}\times d_{c2}}$, $\boldsymbol{W}_{c3}\boldsymbol{\in}\mathbb{R}^{d_{c2}\times d_{c3}}$, $\boldsymbol{W}_{out}\boldsymbol{\in}\mathbb{R}^{d_{c3}\times d_{out}}$ are the weight matrices as parameters; $d_{c1}=$ 300, $d_{c2}=$ 200, $d_{c3}=$ 100 are numbers of neurons for three fully connected neural network layers; $d_{out}$ is the number of sample classes in classification tasks; $\boldsymbol{B}_{c1}$, $\boldsymbol{B}_{c2}$, $\boldsymbol{B}_{c3}$ are bias terms; Flatten function is a flatten layer that collapses the input; RELU is an activation function; softmax is a normalized exponential function; ${dropout}_{c}$ is a dropout neural network layer with a probability of *c*; $\hat{Y}$ is the probability for each label.

## Supplementary Note 5: Biological interpretability module

To comprehend Pathformer’s decision-making process, we used averaging attention maps in row-attention to represent the contributions of different modalities, and SHAP value to decipher the important pathways and their key genes. Finally, the hub module of the updated pathway crosstalk network represents the most critical regulatory mechanism in classification.

***Contribution of each modality***

In Pathformer, row-attention is used to facilitate information interaction between different modalities, that is, row-attention map can represent the importance of each modality. According to the trained model, we obtained row-attention maps of 8 heads in 3 blocks for each sample. For the contribution of each modality, we first integrated all matrices of row-attention maps into one matrix by element-wise average. Then, we averaged this average row-attention matrix along with columns as the attention weights of modalities, i.e., the contribution of modalities. The calculation is as follows:

$$\boldsymbol{A}_{aver}=\frac{1}{N}\sum_{n=1}^{N} \frac{1}{BL}\sum_{b=1}^{BL} \frac{1}{H}\sum_{h=1}^{H} {softmax([{[\boldsymbol{A}_{2}^{(h)}]}^{(b)}]}^{(n)})$$

${attention weight}_{i}=\frac{1}{D_{p}}\sum_{j=1}^{D_{p}} a_{ij}$, $a_{ij}$ is the *i*th row and the *j*th columns of $\boldsymbol{A}_{aver}$

, where *N* is the number of samples, *BL* is the number of blocks, *H* is the number of heads, softmax is a normalized exponential function, and ${attention weight}_{i}$ is the attention weight of dimension *i* of pathway embedding.

***Important pathways and their key genes***

We calculated SHAP values of the gene embedding and the pathway embedding encoded by Transformer module corresponding to each sample and each category, denoted as $\boldsymbol{S}_{gn}^{(j)}\in\mathbb{R}^{D_{p}}$ and $\boldsymbol{S}_{pn}^{(j)}\in\mathbb{R}^{D_{p}}$ respectively. The SHAP values of genes and pathways are calculated as follows:

$$\mathrm{SHAP}_{g}=\sum_{j=1}^{d_{out}} \sum_{e=1}^{D_{p}} \frac{1}{N}\sum_{n=1}^{N} \left| s_{gne}^{(j)} \right|, s_{gie}^{(j)}\in\boldsymbol{S}_{gi}^{(j)}$$

$$\mathrm{SHAP}_{p}=\sum_{j=1}^{d_{out}} \sum_{e=1}^{D_{p}} \frac{1}{N}\sum_{n=1}^{N} \left| s_{pne}^{(j)} \right|, s_{pie}^{(j)}\in\boldsymbol{S}_{pi}^{(j)}$$

, where $g=1,2,\cdots,N_{g}$is the *g*th gene, $g=1,2,\cdots,N_{p}$ is the *p*th pathway, $n=1,2,\cdots,N$ is the *n*th sample, $e=1,2,\cdots,D_{p}$ is dimension *e* of pathway embedding, and $j=1,2,\cdots,d_{out}$ is the *j*th category of sample.

In addition, the z-score of SHAP values of different modalities for each pathway and gene can demonstrate modal complementarity at the gene level and the pathway level, described as follows:

$$\mathrm{SHAP}_{gi}=\sum_{j=1}^{d_{out}} \sum_{e={e_{1}+\cdots+e}_{i-1}}^{e_{i}} \frac{1}{N}\sum_{n=1}^{N} \left| s_{gne}^{(j)} \right|, s_{gie}^{(j)}\in\boldsymbol{S}_{gi}^{(j)}$$

$$\mathrm{SHAP}_{pi}=\sum_{j=1}^{d_{out}} \sum_{e={e_{1}+\cdots+e}_{i-1}}^{e_{i}} \frac{1}{N}\sum_{n=1}^{N} \left| s_{pne}^{(j)} \right|, s_{pie}^{(j)}\in\boldsymbol{S}_{pi}^{(j)}$$

, where $i=1,\cdots,m$ is the *i*th modality, $e_{i}$ is the length of gene embedding and pathway embedding for modality *i*.

Finally, pathways with the top 15 SHAP values in the classification task are considered as important pathways. For each pathway, genes with top 5 SHAP values are considered as the key genes. The core modality on which one gene depends indicates that the SHAP value of that gene ranks higher on this modality than on the others.

***Hub module of the updated pathway crosstalk network***

The calculation of the sub-network score can be divided into four steps: average pathway crosstalk network matrix calculation, network pruning, sub-network boundary determination, and score calculation. First, according to the trained model, the updated pathway crosstalk network corresponding to each sample was given. For average pathway crosstalk network matrix, we integrated all updated pathway crosstalk network matrixs into a matrix by element-wise average and normalization, calculated as follows:

$$\boldsymbol{P}_{up}=min-max(\frac{1}{N}\sum_{n=1}^{N} \boldsymbol{P}_{n}^{'})$$

, where $n=1,2,\cdots,N$ is the *n*th sample, $\boldsymbol{P}_{n}^{'}$ is the updated pathway crosstalk network matrix of the *n*th sample, min-max is the min-max normalization function.

Then, we performed network pruning, that is, removed larger pathway nodes which contains more than 100 genes in the network. This was done to control the size of the sub-network and avoid evaluation bias caused by excessive emphasis on larger pathways. After network pruning, the average pathway crosstalk network matrix is denoted as $\boldsymbol{P}_{up}^{'}$.

Next, we defined the sub-network corresponding to each node and its boundary. When the corresponding element of average pathway crosstalk network matrix exceeds a boundary threshold, we defined that there is a link between two pathways, while otherwise there is no link. The boundary threshold is defined according to the data distribution, which is 99.7% quantile of $\boldsymbol{P}_{up}^{'}$ in TCGA datasets and 99.9% quantile of $\boldsymbol{P}_{up}^{'}$ in liquid biopsy datasets. Sub-networks are defined as follows:

$${{pathlist}_{1}=[p_{11},p_{12},\cdots,p_{1j},\cdots,p_{1r}],P}_{up}^{’}(p_{give},p_{1j})>cut off$$

$${pathlist}_{2}=[p_{21},p_{22},\cdots,p_{2j},\cdots,p_{2r}],\sum_{p_{1j}\in{pathlist}_{1}} {sum(P}_{up}^{'}(p_{2j},p_{1j})>cut off)>0$$

$${SP}_{give}\left( p_{2i},p_{2j} \right)=\left\{ \begin{matrix} P_{up}^{'}\left( p_{2i},p_{2j} \right), if P_{up}^{'}\left( p_{2i},p_{2j} \right)>cut off \\ 0,if P_{up}^{'}\left( p_{2i},p_{2j} \right)\leq cut off \end{matrix} \right.,{p_{2i} and p}_{2j}\in{pathlist}_{2}$$

, where $p_{give}$ is the given pathway as central node, *cut off* is the boundary threshold, ${pathlist}_{1}$ is the list of pathways as neighbours of central node, ${pathlist}_{2}$ is the list of pathways of sub-network, and $\boldsymbol{SP}_{give}$ is the adjacency matrix of sub-network.

Then, we calculated sub-network score as the average of SHAP values of all pathways in the sub-network, which is formulated as:

$$\mathrm{score}_{sub}=\frac{1}{\mathrm{len}({pathlist}_{2})}\sum_{p_{2j}\in{pathlist}_{2}} \mathrm{SHAP}_{p_{2j}}$$

Finally, we defined the sub-network with the highest score as the hub module of the updated pathway crosstalk network.

## Supplementary Note 6: Details of model training and test

In this study, we implemented Pathformer’s network architecture using the “PyTorch” package in Python v3.6.9 (codes in https://github.com/lulab/Pathformer). For model training and test, we used 2 times 5-fold cross-validation. We implemented model training, hyperparameter optimization and model early stopping on the training set (80%) and tested model on the test set (20%).

When training the model, we applied cross-entropy loss with class-imbalance weight as the label prediction loss, the ADAM optimizer to train Pathformer, and the cosine annealing learning rate method to optimized learning rate. "ADAM optimizer" is implemented by “Adam” function in the “PyTorch” package. The cosine annealing learning rate method is implemented by “CosineAnnealingWarmupRestarts” function in the “PyTorch” package, with the first cycle step size as 15, the cycle step magnification as 2, the number of warmup steps as 5, the decrease rate of learning rate by cycle as 0.9, the minimum of learning rate as 1e-8, and the maximum of learning rate as optimal value of hyperparameter optimization.

For hyperparameter optimization, we used grid search with 5-fold cross-validation in the training set with the macro-averaged F1 score as the selection criterion. During grid search process, model was trained for 30 epochs. The key hyperparameters of Pathformer are maximum of learning rate (*lr_max*) $\in$[1e-4, 1e-5], dropout probability of classification (*c*)$\in$[0.3, 0.5], and constant coefficient for row-attention ($\beta$)$\in$[0.1,1], a total of 8 possible combinations. **Supplementary Fig. 3** shows examples of grid search on breast cancer dataset. **Supplementary Table 2** lists results of optimal hyperparameter combination for each dataset. We implemented hyperparameter optimization process of each TCGA dataset for benchmark testing. In addition, we directly set hyperparameters (*lr_max*=1e-5, *c*=0.3, $\beta$=1) of liquid biopsy datasets for application.

To validate the convergence of Pathformer, we depicted the training loss related to the number of epochs (**Supplementary Fig. 4**). In both TCGA datasets and liquid biopsy datasets with different sample sizes and inputs, the total loss rapidly decreased with iteration and eventually converged to a stable state. To prevent overfitting, we employed an early stopping strategy to determine epoch numbers. The early stop strategy refers to stopping training when the macro-averaged F1 score of the validation set (20% of the training set) consecutively decreased more than 1e-2 on 10 epochs. Then we took the model on the epoch before decline as the final model. Additionally, based on the convergence analysis on the liquid biopsy datasets, we set the training epochs to be greater than 100.

## Supplementary Note 7: Comparison methods

For benchmarking, we compared three types of multi-modal integration methods: early and late integration methods based on base classifiers, supervised methods in mixOmics, and deep learning-based integration methods. For deep learning-based integration methods, we benchmarked eight representative models, i.e., eiNN, liNN, eiCNN, liCNN, MOGONet, MOGAT, P-NET and PathCNN.

### *Early integration methods based on base classifiers*

Early integration methods based on base classifiers refer to methods that splice different modal data and perform classification by support vector machine (SVM), logistic regression (LR), random forest (RF), or gradient boosting tree (XGBoost). Specifically, we first uniformly transform the different modalities to the gene level modal features by a conversion function. For each modal feature, we selected the top 1000 genes with FDR$\leq$0.05 of the ANOVA as marker genes, which is implemented by “scikit-learn” package of *Python* v3.6.9. When there were less than 200 genes with FDR<=0.05 of ANOVA, we used P-value instead of FDR. When there were less than 20 genes with P-value <= 0.05 of ANOVA, we only selected genes by their rank. For *j*th modal feature, the number of marker genes is $N_{j}$. Then, we concatenated filtered modal features to obtain features with $N_{gs}=\sum_{j=1}^{D_{g}} N_{j}$ dimensions as the input of the base classifier. Next, to be consistent with Pathformer, we divided the dataset into the training set (80%) and the test set (20%) hierarchically, and performed 5-fold cross-validation on the training set for hyperparameter optimization. SVM was implemented by “SVC” function with kernel=‘rbf’ and C$\in$[0.01, 0.1, 1, 10, 100] in “scikit-learn” package. LR was implemented by “LogisticRegression” function with solver=‘liblinear’, penalty=‘l2’ and C$\in$[0.01, 0.1, 1, 10, 100] in “scikit-learn” package. RF was implemented by “RandomForestClassifier” function with max_depth$\in$[10, 50, 100, 200, 500] and n_estimators$\in$[10, 50, 100, 200, 500] in “scikit-learn” package. XGBoost was implemented by “XGBClassifier” function with learning_rate=0.5, min_child_weight=3, gamma=3, subsample=0.7, scale_pos_weight=1, objective$\in$[‘binary: logistic’, ‘multi: softprob’], max_depth$\in$[10, 50, 100, 200, 500] and n_estimators$\in$[50, 100, 200, 500] in “XGBoost” package.

### *Late integration methods based on base classifiers*

Late integration methods based on base classifiers refer to using SVM, LR, RF, and XGBoost to calculate the classification probabilities of different modality data and take the average probability for prediction. Specifically, we performed preprocessing and marker gene selection on each modality data as described in early integration methods based on base classifiers. We took modal features corresponding to each modality as the input of the base classifier, that is, we established *m* classifiers corresponding to encoded features of *m* modalities. Finally, we took the average classification probabilities of m classifiers as the predicted probability. Here, data partitioning, hyperparameter optimization and the implementation of base classifiers are consistent with early integration methods based on base classifiers.

### *Supervised methods in mixOmics*

The supervised methods in mixOmics refer to partial least squares-discriminant analysis (PLSDA) and sparse partial least squares-discriminant analysis (sPLSDA). PLSDA uses discriminant analysis to project data into latent structures, aiming to find common information across multi-modal data and differentiate between different phenotype groups. sPLSDA is PLSDA appended with sparse regularization. We implemented the PLSDA module and the sPLSDA by the “mixOmics” package of *R* software. Specifically, we performed preprocessing and marker gene selection on each modality data as described in ‘Early integration methods based on base classifiers’ section. Then gene embedding corresponding to each modality were used as input of PLSDA and sPLSDA for model training and testing. In addition, to be consistent with other models, we also divided the dataset into the training set (80%) and the test set (20%) hierarchically, and performed 5-fold cross-validation on the training set to optimize the number of components. The value range of the number of components is [2, 5, 10].

### *eiNN and liNN*

eiNN and liNN are early and late integration methods based on fully connected neural network (FCNN). FCNN usually consists of an input layer, multiple hidden layers, and an output layer. eiNN means flattening all modal features (each dimension of gene embedding) and concatenating them into a vector as input for the neural network. liNN means taking modal features corresponding to each modality as separate inputs to the sub neural network, then connecting the output layers together, followed by a fully connected layer for the final output. We used the code on DL-mo’s Github library (https://github.com/zhenglinyi/DL-mo) to implement models. For eiNN, we set 3 hidden layers with dimensions of 500, 100, and 50, dropout rate as 0.1, and lr as 1e-5. For liNN, we set 3 subnetworks with 2 hidden layers of 100 and 50 dimensions, classification layer with 3 hidden layers dimensions of 100, 50, and 10, dropout rate as 0.1, and lr as 1e-5. Specifically, we performed preprocessing and marker gene selection on each modality data as described in early integration methods based on base classifiers. For model training and test, we divided each dataset into the training set (80%) and the test set (20%) hierarchically, and used 20% of the training set as the validation set to achieve early stopping of the model. When the number of epochs was more than 200 and the macro-averaged F1 score of the validation set consecutively decreased more than 1e-2 on 10 epochs, we stopped training and took the model on the epoch before decline as the final model.

### *eiCNN and liCNN*

eiCNN and liCNN are early and late integration methods based on convolutional neural network (CNN). eiCNN means flattening all modal features and concatenating them into a vector as input for CNN. liCNN means taking modal features corresponding to each modality as separate inputs to each CNN, then connecting the output layers together, followed by a fully connected layer for the final output. We used the code on DL-mo’s Github library (https://github.com/zhenglinyi/DL-mo) to implement models. For eiCNN, we set 2 CNN layers with kernel size of 1000 and 50, 2 maximum pooling layers with size of 100 and 10, fully connected layer with dimensions of 50, and lr as 1e-5. For liCNN, we set 3 subnetworks with a CNN layer and a maximum pooling layer, classification layer with 3 hidden layers dimensions of 100, 50, and 10, dropout rate as 0.1, and lr as 1e-5. In addition, we performed preprocessing, marker gene selection on each modality data, data partitioning, and early stopping strategy of eiCNN and liCNN are consistent with eiNN and liNN as described above.

### *MOGONet and MOGAT*

MOGONet model first uses graph convolution to learn weighted sample similarity network and the matrix of a single modality, and then uses the View Correlation Discovery Network (VCDN) to integrate the classification probabilities of different modalities. MOGAT model builds upon MOGONet by replacing the graph convolutional neural network with a graph attention neural network. We used the code on MOGONet’s Github library (<https://github.com/txWang/MOGONET>) to implement MOGONet model and DL-mo’s Github library (https://github.com/zhenglinyi/DL-mo) to implement MOGAT model. We set num_epoch_pretrain as 200, adj_parameter as 2, dim_he as 100, lr_e_pretrain as 1e-3, lr_e as 5e-4, lr_c as 1e-3, and lr as 1e-5 respectively. We then took modal features corresponding to each modality as the input of MOGONet for downstream analysis. In addition, we performed preprocessing, marker gene selection on each modality data, data partitioning, and early stopping strategy of MOGONet and MOGAT are consistent with eiNN and liNN as described above.

### *P-NET*

P-NET model is a sparse neural network integrating multiple molecular features based on a multilevel view of biological pathways. In P-NET, all dimensions of gene multi-modal embedding are connected together as inputs, and then distributed on node layers representing a set of genes using weighted links. The other hidden layers of P-NET are constructed based on the hierarchical structure of pathways. The connections between different layers are limited to the child-parent relationship between features, genes, and pathways. In particular, P-NET does not need feature selection. We rewrote the code based on the PyTorch following the code in the P-NET’s Github library (https://github.com/marakeby/pnet_prostate_paper) to implement the model, and set pathway dataset to Reactome dataset, n_hidden_layers as 5, activation as ‘tanh’, kernel_initializer as ‘glorot_uniform’, bias_initializer as ‘zeros’, batch_normal as ‘False’, repeated_outcomes as ‘True’, dropout as [0.5, 0.1, 0.1, 0.1, 0.1, 0.1, 0.1], and lr as 1e-5. In addition, data partitioning and early stopping strategy of P-NET are consistent with eiNN as described above.

### *PathCNN*

PathCNN model is a classic model that introduces known pathway knowledge for multi-modal integration. PathCNN first uses principal component analysis (PCA) to integrate multi-modal data into the pathway level as pathway images of multi-modal, and then uses convolutional neural network (CNN) to extract high-dimensional features for downstream classification tasks. We used the code on PathCNN’s Github library (https://github.com/mskspi/PathCNN) to implement the model, and set lr to 1e-5. Specifically, we first uniformly transform the different modalities to the gene level modal features by a conversion function. Then, we filtered the modal features corresponding to genes of 146 pathways in PathCNN, and used the PCA to integrate these to obtain multi-modal pathway images. Finally, we took pathway image of each modality as input to PathCNN for downstream analysis. In addition, data partitioning and early stopping strategy of PathCNN are consistent with eiNN and liNN as described above.

## Supplementary Note 8: Reliability analysis of Pathformer’s confidence scores

To analyze the reliability of Pathformer, we depicted confidence score histograms and calibration curves of Pathformer on multiple TCGA datasets and the liquid biopsy datasets (**Supplementary Fig. 5**). The confidence score histogram displays the distribution of samples across various confidence score intervals. It provides a visual representation of the model’s performance at different confidence levels, offering deeper insights into its behavior. The calibration curve, also known as the reliability diagram, depicts the relationship between the model’s predicted confidence scores and frequency of the positive label. Here, we binned confidence scores from 0 to 1 into 10 intervals with width 0.1 each to generate confidence score histograms and reliability diagrams for Pathformer across different datasets. Please note that all samples from each dataset were used to ensure sufficient sample size for confidence score binning in **Supplementary Fig. 5**. From the results of confidence score histograms, Pathformer show balance property in most datasets, similar to logistic regression. In other words, in Pathformer, most samples’ confidence scores tend towards 0 or 1, but others uniformly distribute in other intervals. This is likely because Pathformer uses the softmax function to transform confidence scores in the final layer. From the results of calibration curves, Pathformer’s prediction accuracy generally correlates positively with confidence scores, although not linearly. In other words, confidence score is a good estimate of prediction accuracy in Pathformer. Next, we depicted various accuracy metrics (accuracy, F1score_macro, precision, and recall) at different thresholds of Pathformer's confidence scores across various datasets, further substantiating the validity of Pathformer's confidence scores (**Supplementary Fig. 6**). Similar to calibration curves, we select confidence scores at intervals of 0.1 as thresholds, predicting samples below these thresholds as negative and above as positive, to calculate various accuracy metrics. We observed that as the threshold increased, precision continuously rose, recall gradually declined, and accuracy initially increased before stabilizing. Additionally, to compare the reliability of Pathformer with other integrated models, we computed pearson correlation coefficients between confidence scores (x-axis of reliability diagram) and fraction of positives (y-axis of reliability diagram) across each dataset’s test sets (**Supplementary Fig. 7**). The results indicate that Pathformer not only demonstrates higher average correlation compared to other methods but also maintains relatively stable performance across different datasets. In conclusion, Pathformer demonstrates better reliability compared to other integrated methods.

## Supplementary Note 9: Correct predictions analysis

In previous analyses, we used Pathformer’s attention weights and SHAP values to identify relevant pathways, genes, and patterns for classification, discussing their alignment with established biological literature (**Fig. 4-5**). To gain further insights into how Pathformer utilizes these features for accurate decision-making, we attempted to visualize pathway embedding changes and explored commonalities among correctly classified samples. First, we compared the principal component analysis (PCA) of pathway embedding matrices before and after Pathformer update on BRCA survival risk classification and stage classification (**Supplementary Fig. 12a and Supplementary Fig. 13a**). While the initial pathway embedding matrix showed poor classification performance, the updated matrix after Pathformer adjustments notably grouped samples into two distinct classes, correctly categorizing most samples and misclassifying a small fraction. This suggests that Pathformer efficiently extracts features for classification by Transformer module with CC-attention, enabling each updated pathway to encapsulate not only its own information but also insights from other pathways, particularly those with crosstalk. Next, to visually explore commonalities among correctly classified samples, we generated pathway score heatmaps for active pathways on BRCA survival risk classification and stage classification (**Supplementary Fig. 12b and Supplementary Fig. 13b**). We can observe that the performance of incorrectly classified samples in these pathways often differs from their true classes, especially in BRCA survival risk classification. For example, incorrectly classified high-risk breast cancer patients showed lower scores in the *FRA* pathway and higher scores in the *interaction between L1 and ankyrins* pathway, resembling actual high-risk patients. In BRCA stage classification, although pathway score patterns were less prominent, distinctions between the two classes and incorrectly classified samples are still noticeable, especially in the highlighted regions of **Supplementary Fig. 13b**. Subsequently, we attempted to identify whether correctly classified samples shared certain common clinical characteristics. We compared the distribution of subtype and age among correctly classified samples and incorrectly classified samples on BRCA survival risk classification and stage classification (**Supplementary Fig. 12c-d and Supplementary Fig. 13c-d**). The chi-square tests revealed no significant impact of subtype and age on Pathformer’s prediction results. Pathformer does not exhibit bias towards patients with certain clinical characteristics, demonstrating its robustness.

Additionally, we conducted a Pathformer decision process analysis on plasma and platelet data, with conclusions similar to those in breast cancer-related classification tasks (**Supplementary Fig. 19-20**). We found that the pathway embedding matrix updated by Pathformer effectively distinguishes between healthy individuals and cancer patients, with distinct patterns of pathway scores observed in each group. We observed that clinical factors such as gender and age had no significant impact on Pathformer’s prediction results in liquid biopsy datasets, while cancer type did. However, this conclusion needs further validation with more samples, given Pathformer’s low misclassification rate in liquid biopsy datasets, which leads to a scarcity of samples for most cancer types in incorrectly classified samples (e.g., the plasma data includes 1 STAD, 2 CRC, 2 HCC, 3 LUAD, and 7 ESCA, while the platelet data includes 1 CRC, 2 BRCA, 8 GBM, and 18 LUAD).

To further understand Pathformer’s commonalities across similar tasks, we took breast cancer survival risk classification and stage classification as examples, examining the intersection of active pathways and the distribution of clinical indicators in correctly classified samples in both tasks (**Supplementary Fig. 14**). **Supplementary Fig. 14a** shows a positive correlation between pathway importance in BRCA survival risk classification and stage classification. Moreover, key pathways like *complex I biogenesis* pathway, *ATP formation by chemiosmotic coupling* pathway, *mitochondrial translation* pathway, and *iron uptake and transport* pathway are vital for Pathformer’s accurate predictions in both tasks, linked to cancer cell energy metabolism and proliferation(Cairns, et al., 2011; Stine, et al., 2022; Torti and Torti, 2013; Zou, et al., 2016). **Supplementary Fig. 14b** indicates that samples correctly classified in both tasks show no significant preferences in age and subtype, suggesting Pathformer’s robustness in both tasks.

To explore whether Pathformer can could reveal additional patterns beyond classification tasks, we took the pan-cancer dataset from TCGA for survival risk classification and staging classification as examples, clustering samples based on key biomarkers in both tasks. First, we present the intersection of active pathways in the two tasks, totaling 45 pathways, such as *complex I biogenesis* pathway (**Supplementary Fig. 15a**). Next, we dimensionally reduced the initial pathway embedding matrix of these 45 active pathways and found that samples primarily clustered by tissue type (**Supplementary Fig. 15b**). Subsequently, we separately employed dimensionality reduction on active pathway embedding matrices updated by Pathformer for pan-cancer survival risk and staging classification (**Supplementary Fig. 15c**). This analysis revealed that samples mainly clustered based on classification label and tissue type, with clinical indicators such as age and gender having no impact on the clustering results. In other words, Pathformer primarily uncovers patterns related to classification tasks by feature extraction while retaining the original tissue pattern of the samples, without yet discovering other potential patterns.

## Supplementary Note 10: Feature selection for cancer noninvasive diagnosis

To understand the necessity of liquid biopsy multi-modal integration, we first calculated seven RNA-level modalities as Pathformer’s input separately. From results of 2times 5-fold cross-validation in **Supplementary Fig. 16**, we found that the model with all modalities as input had the best comprehensive performance on two datasets, followed by RNA expression-only model and RNA alt. promoter-only model, and some models with other modalities exhibited great fluctuations on different datasets. In order to effectively integrate information without redundancy, we performed further feature selection based on different modality combinations evaluated by Pathformer. First, we calculated the contributions of each modality and its corresponding statistical indicators (**Supplementary Fig. 17a**). Similar to the results of cross-validation, RNA expression was the core modality across all datasets. Next, we performed 5-fold cross-validation find an optimal modality combination for each dataset (**Supplementary Fig. 17b**). We found that plasma dataset with 7 modalities and platelet dataset with 3 modalities (RNA expression, RNA alternative promoter, and RNA splicing) obtained the best performance.

## Supplementary Note 11: Explained variances of different models and modalities

To effectively demonstrate Pathformer’s capability in interpreting multi-modal biological data, we compared its explanation variance with 18 other integration models on both the TCGA datasets and liquid biopsy datasets (**Supplementary Fig. 21a-b**). Explanation variance, a crucial measure of a model’s ability to explain observed data variability, is typically measured in deep learning by comparing the variance between model predictions and actual labels in the test set. Despite typically lower interpretability in deep learning models, Pathformer demonstrated competitive explanation variance, particularly excelling in the liquid biopsy data. This result signifies Pathformer’s ability to understand and explain complex multi-omics data, validating its potential for real-world clinical applications. Furthermore, we compared the explanation variance across different modalities in the TCGA datasets and the liquid biopsy datasets (**Supplementary Fig. 21c-d**). The TCGA dataset involves three modalities, whereas the liquid biopsy dataset includes seven. We calculated the explanation variance for each modality separately by using them as inputs to Pathformer. The results indicate that the explanation variance in different modalities corresponds to their predictive performance, with RNA expression data notably outperforming other modalities. We calculated the explanation variance for each modality separately by using them as inputs to Pathformer. The results indicate that the explanation variance in different modalities corresponds to their predictive performance, with RNA expression data notably outperforming other modalities. Notably, the TCGA dataset’s three modalities show more consistent explanation variance, while in the liquid biopsy data, there’s a greater variance difference between modalities. This finding underscores the importance of feature selection in analyzing liquid biopsy data.

## Supplementary Note 12: Pathformer’s resource and time consumption

To promote the application of Pathformer, we used BRCA survival risk classification as an example and compared the resource and time consumption of Pathformer with other models (**Supplementary Table 5**). We observed that Pathformer requires more memory and computational time compared to other models, primarily due to two reasons. Firstly, Pathformer does not perform additional feature selection and utilizes all pathways and 11,560 mRNAs from four common databases, while most other algorithms, except Pnet, employed feature selection (**Supplementary Note 7**). Avoiding additional feature selection helps prevent the loss of potentially relevant information but leads to managing more features, necessitating the processing of larger data matrices and thus requiring greater memory allocation. Secondly, Pathformer is a Transformer-based deep learning model with longer inputs. Although its pathway embedding prevents memory overflow in the Transformer module caused by long inputs, its input length of 1498 still necessitates substantial space and time for training. While Pathformer demands more time and resources for training its complex model structure, this investment is made to achieve more accurate, stable, and reliable prediction outcomes. Moreover, in the testing phase, Pathformer’s processing time is comparable to other models, averaging 1-2 seconds, effectively meeting the clinical requirements for quick analysis of new samples with trained models. Overall, although Pathformer consume more resources and time during training, its efficacy and reliability make it a preferred tool in research and practice, offering robust support and guidance to scientists and clinicians.

# References

6 7 Lehmann Kjong-Van 3 4 6 7 Liu Fenglin 10 Shiraishi Yuichi 11 Soulette Cameron M. 12 Urban Lara 2, P.T.C.G.C.C.D.N.R.D.D.F.N.A.H.Y.K.A., et al. Genomic basis for RNA alterations in cancer. Nature 2020;578(7793):129-136.

Best, M.G., et al. Swarm intelligence-enhanced detection of non-small-cell lung cancer using tumor-educated platelets. Cancer cell 2017;32(2):238-252. e239.

Best, M.G., et al. RNA-Seq of tumor-educated platelets enables blood-based pan-cancer, multiclass, and molecular pathway cancer diagnostics. Cancer cell 2015;28(5):666-676.

Cairns, R.A., Harris, I.S. and Mak, T.W. Regulation of cancer cell metabolism. Nature Reviews Cancer 2011;11(2):85-95.

Castel, S.E., et al. Tools and best practices for data processing in allelic expression analysis. Genome biology 2015;16(1):1-12.

Chen, S., et al. Cancer type classification using plasma cell-free RNAs derived from human and microbes. eLife 2022;11:e75181.

Chiu, Y.-C., et al. Predicting drug response of tumors from integrated genomic profiles by deep neural networks. BMC medical genomics 2019;12:143-155.

Ding, Z., Zu, S. and Gu, J. Evaluating the molecule-based prediction of clinical drug responses in cancer. Bioinformatics 2016;32(19):2891-2895.

Harrow, J., et al. GENCODE: the reference human genome annotation for The ENCODE Project. Genome research 2012;22(9):1760-1774.

Lee, M., et al. ChimerDB 3.0: an enhanced database for fusion genes from cancer transcriptome and literature data mining. Nucleic Acids Research 2017;45(D1):D784-D789.

Marques, O., et al. Iron homeostasis in breast cancer. Cancer letters 2014;347(1):1-14.

Picardi, E., et al. REDIportal: a comprehensive database of A-to-I RNA editing events in humans. Nucleic acids research 2017;45(D1):D750-D757.

Schmitt, T., Ogris, C. and Sonnhammer, E.L. FunCoup 3.0: database of genome-wide functional coupling networks. Nucleic acids research 2014;42(D1):D380-D388.

Schneider, V.A., et al. Evaluation of GRCh38 and de novo haploid genome assemblies demonstrates the enduring quality of the reference assembly. Genome research 2017;27(5):849-864.

Sharifi-Noghabi, H., et al. MOLI: multi-omics late integration with deep neural networks for drug response prediction. Bioinformatics 2019;35(14):i501-i509.

Singh, S., et al. The landscape of chimeric RNAs in non-diseased tissues and cells. Nucleic acids research 2020;48(4):1764-1778.

Stine, Z.E., et al. Targeting cancer metabolism in the era of precision oncology. Nature reviews Drug discovery 2022;21(2):141-162.

Tao, Y., et al. Cell-free multi-omics analysis reveals potential biomarkers in gastrointestinal cancer patients’ blood. Cell Reports Medicine 2023;4(11).

Torti, S.V. and Torti, F.M. Iron and cancer: more ore to be mined. Nature Reviews Cancer 2013;13(5):342-355.

Zou, P., et al. Coordinated upregulation of mitochondrial biogenesis and autophagy in breast cancer cells: the role of dynamin related protein-1 and implication for breast cancer treatment. Oxidative medicine and cellular longevity 2016;2016.
